# Principles, developments, and applications of spatially resolved spectroscopy in agriculture: a review

**DOI:** 10.3389/fpls.2023.1324881

**Published:** 2024-01-10

**Authors:** Yu Xia, Wenxi Liu, Jingwu Meng, Jinghao Hu, Wenbo Liu, Jie Kang, Bin Luo, Han Zhang, Wei Tang

**Affiliations:** ^1^ School of Electrical and Control Engineering, Shaanxi University of Science & Technology, Xi’an, Shaanxi, China; ^2^ Intelligent Equipment Research Center, Beijing Academy of Agriculture and Forestry Sciences, Beijing, China

**Keywords:** spatially resolved spectroscopy, optical properties, quality inspection, agriculture, hyperspectral imaging

## Abstract

Agriculture is the primary source of human survival, which provides the most basic living and survival conditions for human beings. As living standards continue to improve, people are also paying more attention to the quality and safety of agricultural products. Therefore, the detection of agricultural product quality is very necessary. In the past decades, the spectroscopy technique has been widely used because of its excellent results in agricultural quality detection. However, traditional spectral inspection methods cannot accurately describe the internal information of agricultural products. With the continuous research and development of optical properties, it has been found that the internal quality of an object can be better reflected by separating the properties of light, such as its absorption and scattering properties. In recent years, spatially resolved spectroscopy has been increasingly used in the field of agricultural product inspection due to its simple compositional structure, low-value cost, ease of operation, efficient detection speed, and outstanding ability to obtain information about agricultural products at different depths. It can also separate optical properties based on the transmission equation of optics, which allows for more accurate detection of the internal quality of agricultural products. This review focuses on the principles of spatially resolved spectroscopy, detection equipment, analytical methods, and specific applications in agricultural quality detection. Additionally, the optical properties methods and direct analysis methods of spatially resolved spectroscopy analysis methods are also reported in this paper.

## 1 Introduction

With the improvement of living standards, people have higher and higher requirements for the quality and safety of agricultural products (Rejeb et al., 2022). Nondestructive testing techniques for the quality of agricultural products have also become more and more widespread in recent years (Tian et al., 2023). With the development of optical technology, some efficient and mature optical nondestructive detection techniques have emerged (Mei and Li, 2023; Mohd Ali et al., 2023), such as visible and near-infrared (Vis-NIR) spectroscopy (Guo et al., 2023) and hyperspectral imaging (HSI) (Chen et al., 2021; Tian et al., 2021; Zhang et al., 2022; Zhao et al., 2023), which have been widely used in nondestructive quantities for physical and chemical characterization of agricultural products. These optical inspection techniques can be mainly used to measure the spectral information of agricultural products to obtain the diffuse reflectance (or transmittance) of the samples and then combine this spectral information with existing chemometrics algorithms to establish a prediction model for the quality of agricultural products. Although existing intelligent information processing techniques are more mature, such as deep learning and machine learning (Audu and Aremu, 2021; Dhanya et al., 2022; Ryo, 2022), these methods have been widely developed and can further enhance the ability to detect the quality of agricultural products. Nevertheless, the spectral information which has already been obtained, can be only analyzed by these methods, and if the spectral information obtained is better, then the quality of agricultural products will be more accurately detected. When light enters the surface of an object, a series of optical phenomena such as scattering and absorption will occur, and this optical information is very important for the detection of the quality of the spectrum. The common spectral acquisition methods often produce significant errors and cannot accurately describe the absorption and scattering information of the light. In order to describe more accurately the laws of propagation of light in the organization of an object as well as more specific properties, special studies have been made on optical properties (OP).

When light enters a turbid medium, a series of optical phenomena occur, such as reflection, refraction, absorption, and scattering. Absorption and scattering of light are the most dominant OP of light in biological tissues. The absorption coefficient (*µ_a_*) and the reduced scattering coefficient (*µ_s_’*) are specific descriptions of the absorption and scattering properties of light. The *µ_a_* is mainly related to the chemical composition of the biological tissue, while the *µ_s_’* is closely associated with the structural and physical properties of the sample tissue. Conventional optical inspection techniques can only detect the total effect of light absorption and scattering, but it is not easy to measure the specific parameters of these OP accurately. Researchers have made great efforts to distinguish between scattering and light absorption effects in tissues. Currently, indirect measurement techniques for optical parameters, represented by time-resolved (TR) (Cubeddu et al., 2001; Zude et al., 2011; Vanolia et al., 2020), spatial-resolved (SR) (Ma et al., 2021b; Huang et al., 2022), frequency-domain (FD) (Hu et al., 2020a), spatial-frequency domain imaging (SFDI) (Hu et al., 2018; Sun Z. et al., 2021) and integrating sphere (IS), are used by measuring intact or partial tissue via obtaining certain specific parameters (such as diffuse reflectance *R*, diffuse transmittance *T*, and collimated transmittance *T_c_*, etc.) of intact tissue or slices and combining them with specific optical transmission models and inversion algorithms, the optical parameters of the sample can be obtained indirectly, and the absorption and scattering properties of tissues from light can be separated or obtained simultaneously, thus the chemical and physical information of sample can be eventually reflected. Compared with other detection techniques towards optical properties, spatially resolved spectroscopy (SRS) is simple, low cost, and is widely used and relatively mature in nondestructive testing of agricultural products.

**Figure 1:**
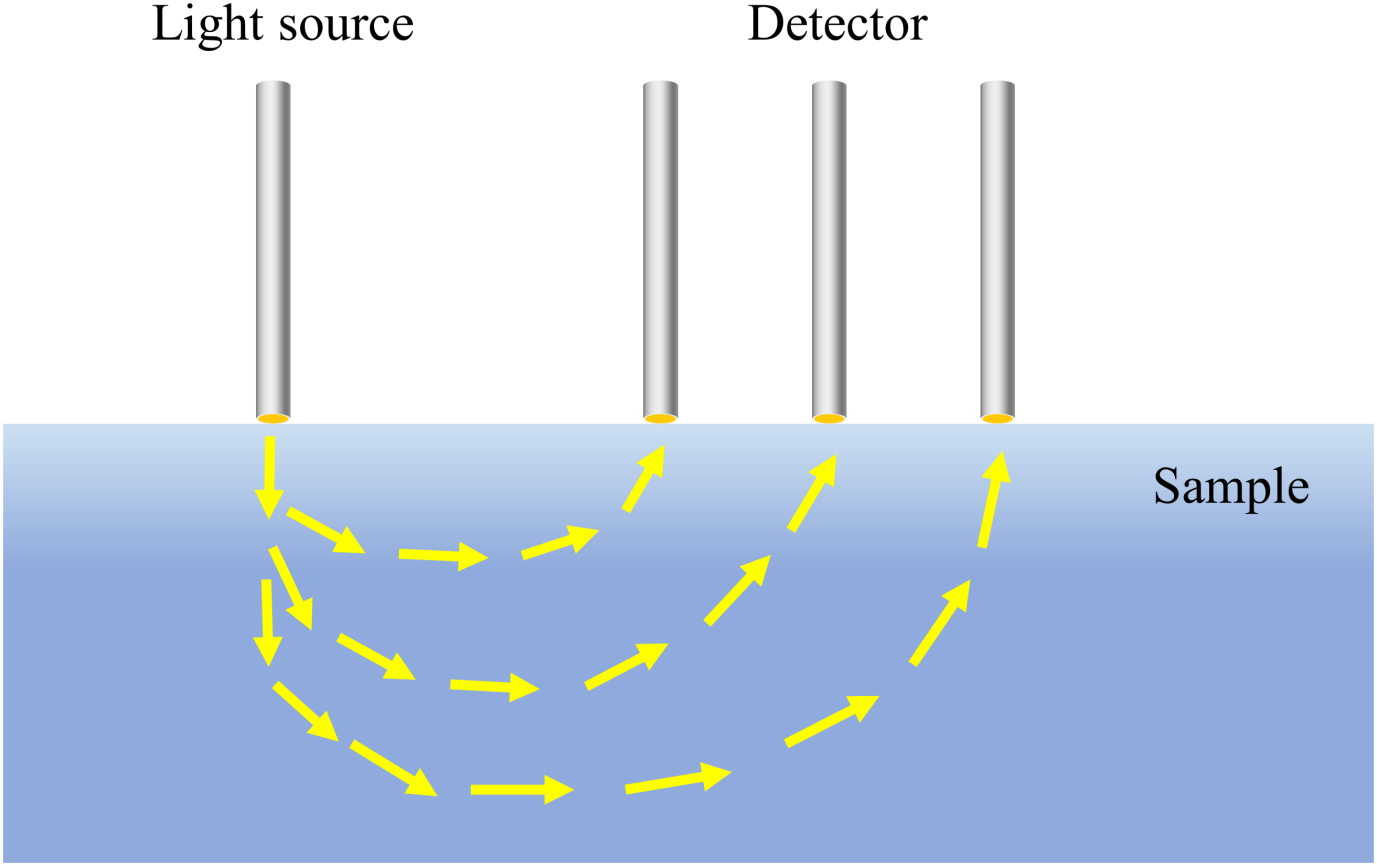
Transmission paths of light.

SRS was initially used in the medical field to determine the absorption and scattering properties of light in blood with two parallel optical fibers (Reynolds et al., 1976). This technique is mainly used to measure the diffuse reflection of light at different distances from the sample surface via a point light source and to calculate the absorption and reduced scattering coefficients of light in biological tissues by combining the diffuse reflection equation of light. It has a banana-shaped transmission path, as shown in **Figure 1**. As the distance between the light source and the detector increases, the SRS method can detect deeper, which can obtain more information about the interior of the corresponding tissue and facilitate the detection of features inside biological tissues. In summary, SRS is a convenient tool for obtaining spectral information at different locations. Since SRS integrates spatial and spectral information, it can help researchers to explore its correlation with the chemical composition, physical structure and OP of the samples to be measured, and to build corresponding prediction models for the purpose of product quality prediction, which has resulted in a wider application of this technology in more fields. For example, agriculture (Huang et al., 2022), forestry (Ma et al., 2021c), industrial construction (Wang et al., 2022), physical and chemical materials (Bao et al., 2021; Liu et al., 2022), astronomical observation (Bao et al., 2021; Comerford et al., 2022), gas detection (Li et al., 2021), biomedicine (Niwayama and Unno, 2021; De Man et al., 2023) and other fields, providing people with crucial scientific basis and reliable data support. In the field of biomedicine, SRS is widely used in human hemoglobin detection, skin pathology detection, and so on. It can help doctors to more accurately identify hemoglobin levels and the human condition so that they can precisely analyze the cause of the patient illness (Zaytsev et al., 2022; Zhang et al., 2023). It can also be used to identify blood species to enhance wildlife protection and preserve national resource information (Zhang et al., 2021). In the field of agriculture, SRS technology is more advantageous than traditional spectral detection technology, and the prediction model established by this technology can improve the prediction ability of the quality for agricultural products, which is currently mainly applied in the quality detection of SSC (Soluble Solids Content), firmness, pH, bruise detection, etc. (Huang et al., 2018b). It can be seen that SRS has a very wide range of utilization in detection with a broad application prospect.

Traditional detection can only obtain the spectrum of a certain place in the sample without gaining more information, and it often collects the total effect of absorption and scattering of light, which may lead to inaccurate prediction results. In contrast, the SRS method can detect spectra at different distances to obtain more spectral information. Moreover, the technique has the advantage of separating the optical properties to analyze the quality of the sample in a targeted manner. Currently, there are many studies based on SRS in the quality inspection of agricultural products, such as the inspection of fruits, meat products, and milk. Since its detection methods establish models that can predict the quality of agricultural products more accurately, it has been widely used in the field of agriculture. There are fewer researchers who have summarized the principles, development, and applications of SRS in agriculture. Therefore, the main objective of this paper is to provide a systematic introduction to different SRS systems and to review the fundamentals, recent developments, and applications of SRS in agricultural quality inspection. In addition, although SRS has been relatively mature in agriculture, it is still faced with many challenges and difficulties presently. The development status and development trend of SRS techniques in agriculture are also reported.

## 2 Spatially resolved spectral detection systems

With the development of SRS technology, the application field has become more widespread. When using this technique to detect different kinds of samples, people find that some traditional test samples are difficult to meet the needs of different varying detection samples. Not only are there irregularities in the tested models, but modern developments are also demanding faster, more convenient, and more efficient detection configurations or systems, as well as higher detection accuracy and lower device costs. Therefore, researchers are constantly researching and developing more appropriate spatially resolved related detection systems. The following are the existing spatially resolved spectral detection systems at this stage, which mainly include single-fiber, fiber-array, charge-coupled device (CCD) line-scan, hyperspectral line-scan, and multi-channel hyperspectral detection systems.

### 2.1 Single fiber system

The earliest form of spatially resolved spectral detection was to detect spectral information from different distances by two parallel optical fibers in contact with the object under test (Reynolds et al., 1976). This approach is known as the single fiber system. Only two optical fibers are needed; one is connected to a light source to provide a stable optical signal, and another is connected to a spectrometer to receive the signal. The two fibers follow a certain distance to obtain spatially resolved spectra. This type of format is the simplest, but a large error still exists; it is hard to ensure that the light source-detector distance (SDD) is accurate as well as stable when the fiber is moving, and the two fibers also must be as close as possible to the object under test, so as to avoid the impact of stray light on the quality of the spectral information.

**Figure 2:**
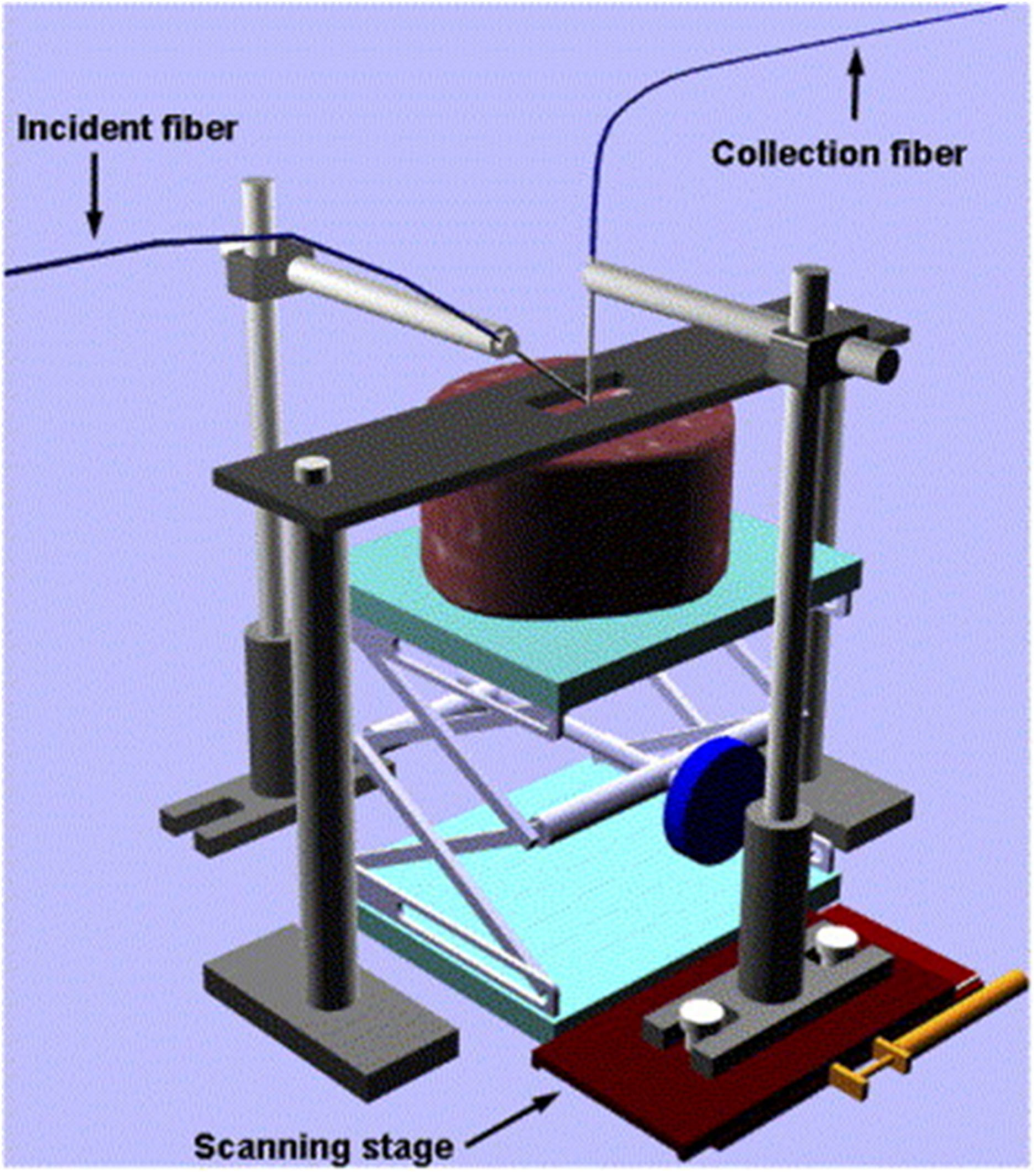
Single fiber optic inspection device (Xia et al., 2007).

To avoid the impact of manual detection on the experiment, Xia et al. fixed the light source fiber and the detection fiber by a mechanical device (Xia et al., 2007), as shown in **Figure 2**, which used a 20 W halogen lamp (HL-2000-FHSA-HP, Ocean Optics Inc., Dunedin, USA) as the light source. It is connected to an optical fiber and illuminates the sample surface at an incidence angle of 40°. The detection fiber, connected to the spectrometer, is perpendicular to the sample surface. The position of the detection fiber is moved by a translation device to detect the spectral information at different distances. Both the source and detection fibers have a core diameter of 400 µm, and the closest distance between the two fibers is 1.5 mm to avoid fiber collisions.

**Figure 3:**
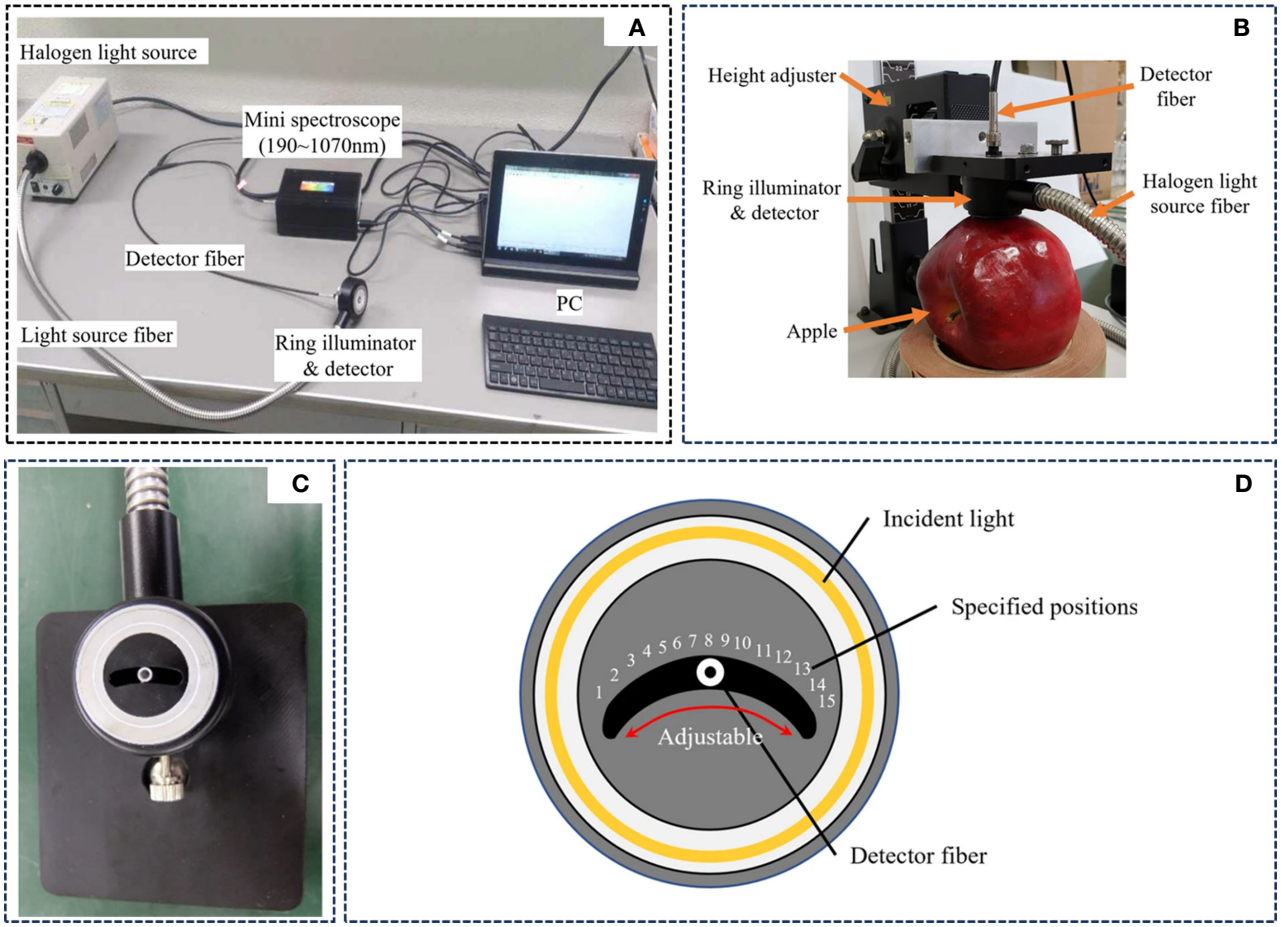
Removable probe fiber optic ring device (Ye et al., 2021). **(A)** Slideable ring inspection system. **(B)** Inspection demonstration image. **(C)** Ring illuminator object diagram. **(D)** Ring illuminator schematic diagram.

For more portability and ease of operation, Ye et al. developed a slidable ring device consisting of a halogen lamp LA-150ue-A (Hayashi Co., Japan), a removable detection fiber ring illuminator, and a Mini-Spectrometer BLACK-Comet-SR100 (StellarNet Inc., USA) (Ye et al., 2021), as shown in **Figure 3A**. Measuring with the ring illuminator close to the surface of the fruit (**Figure 3B**). **Figure 3C** shows a schematic diagram of the ring illuminator. A halogen light source enters the ring illuminator through an optical fiber to form a ring beam, as shown in **Figure 3D**. As the device inside and outside the machine have the effect of shading to reduce the reflection of light from the sample surface, the spectrum is received only through the small hole in the middle of the signal to reduce the impact of mixed spectral information. The detector and light source are in contact with the sample, and the spectral information is detected by moving the position of the detection fiber in the center of the ring.

The single fiber moving detection form is simple in structure, easy to operate, low cost, and flexible. It can select the optimal detection SDD so that the collected information is more representative. However, this method is easily affected by many factors, such as the accuracy and stability of the moving platform, the strength of the light source fiber and the acquisition fiber fixed, the extent of contact between the measured sample irregularities and the acquisition fiber, all of which can make the system have a significant error. In addition, the single fiber detection form has a high demand on the fiber diameter, which requires the fiber diameter to be as thin as possible so that the light SDD can be closer. Xia et al. reduced the light SDD because of the limitation of the fiber diameter adjusted, thereby adjusting the incident light angle (Xia et al., 2007). Furthermore, the time required for single fiber detection is long. Therefore, the use of a single fiber detection format is not friendly for collecting a large number of samples, and a faster and more efficient detection system needs to be developed.

### 2.2 Fiber array type system

**Figure 4:**
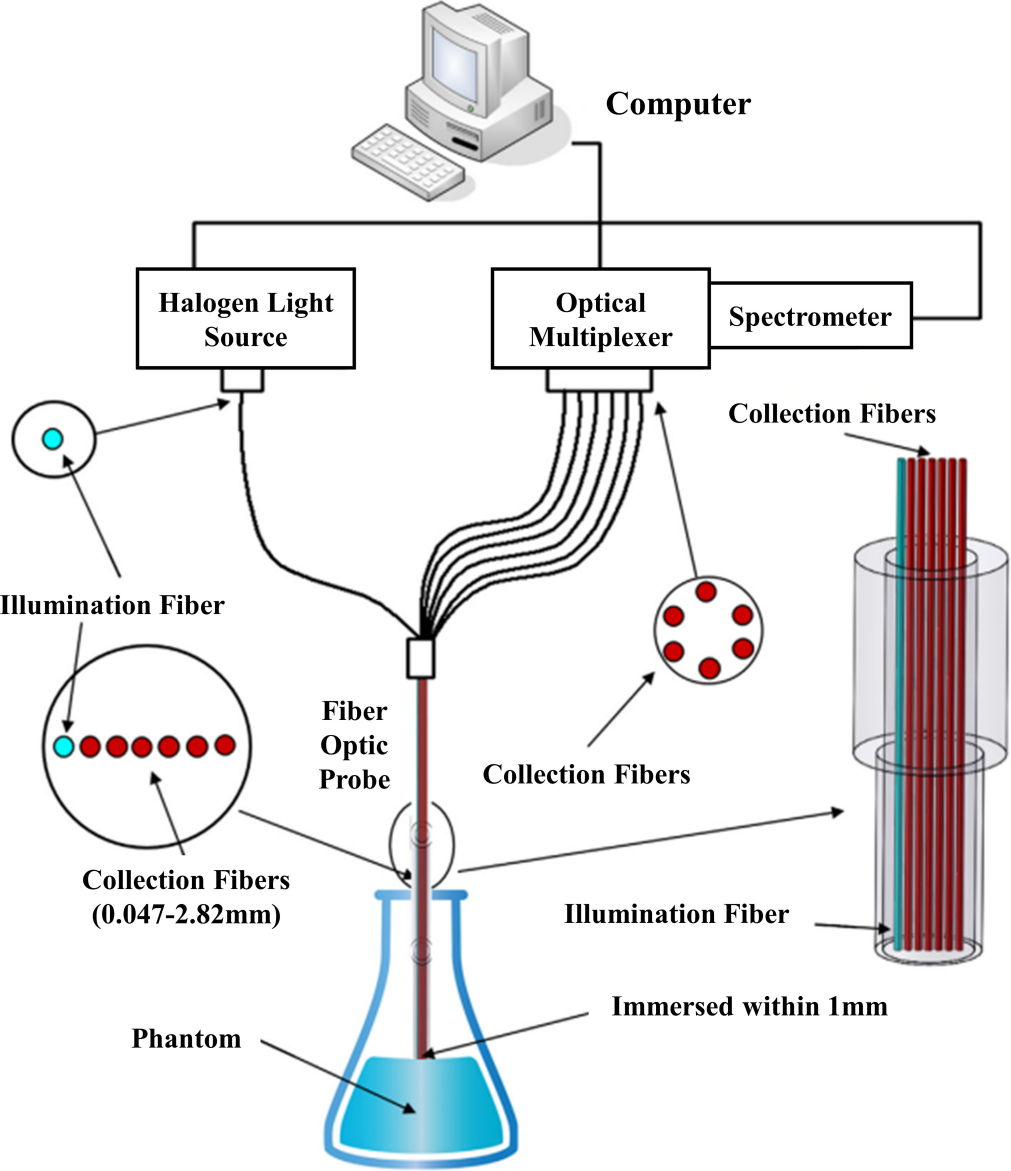
Fiber optic array type inspection device (Zhou et al., 2015).

Due to the significant error of a single fiber optic collection system, the acquisition process of each distance can only follow the sequence to collect, which is time-consuming and laborious, and there will be a phenomenon of missed collection, so the researchers have developed a form of detection based on fiber optic arrays to achieve the simultaneous acquisition of multiple distances. Zhou et al. evaluated the OP of turbid media utilizing a multi-fiber detection format (Zhou et al., 2015), as shown in **Figure 4**. The system was used to collect spatially resolved diffuse reflections at 633 nm with a light source (HL-2000, Ocean Optics, USA), an illumination fiber, six detection fibers, and a spectrometer (QE65pro, Ocean Optics, USA). All the acquisition fibers are connected to a multiplexer, and the spectral signal is transmitted to the spectrometer through the multiplexer.

**Figure 5:**
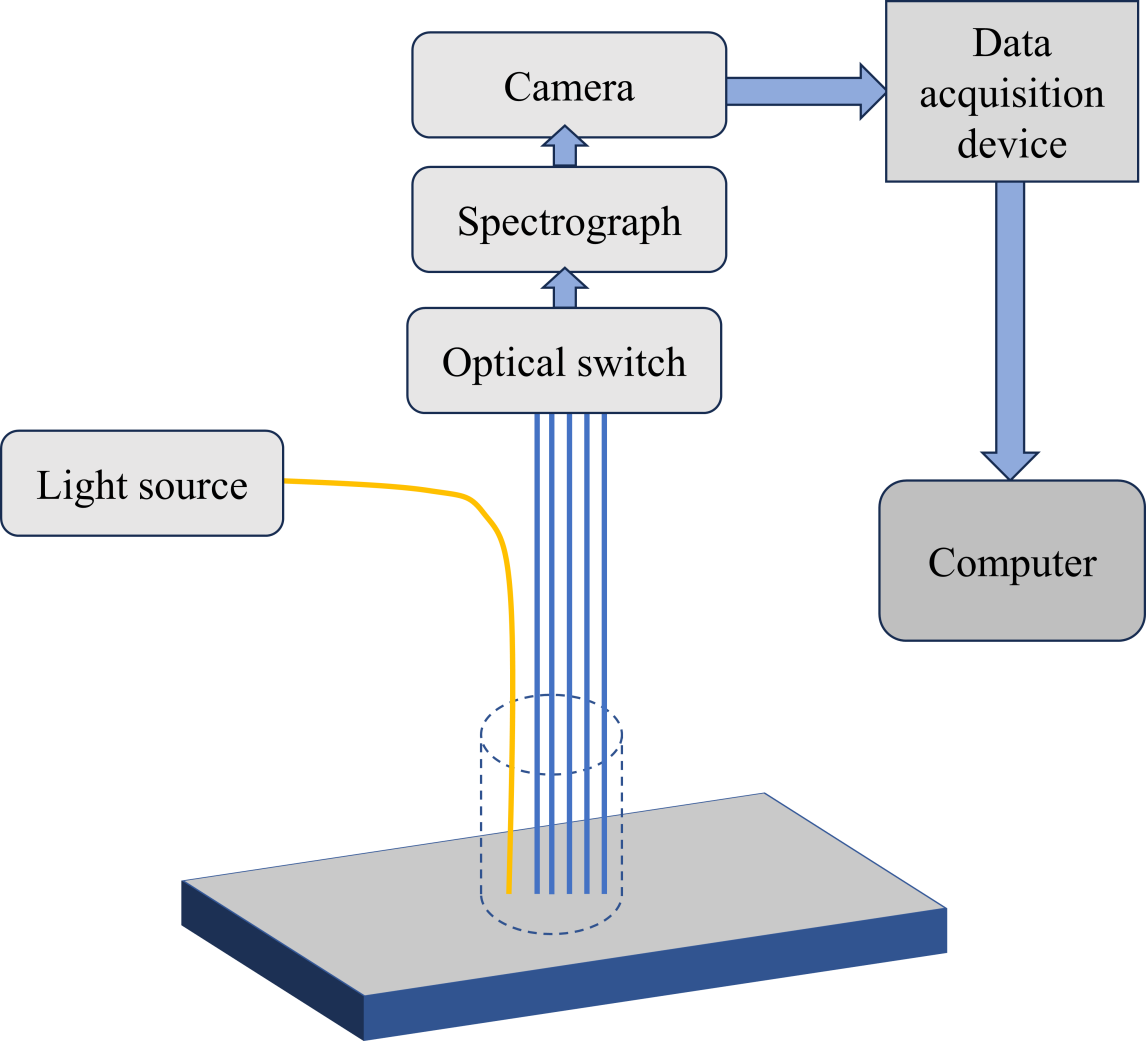
Novel fiber optic array device (Nguyen Do Trong et al., 2011).

Spectral information for every distance can be read by a fiber array device connected to the multiplexer. Nevertheless, the sequential reading of each spectral information needs to be set up, and the setup is complicated with a longer reading time. Nguyen Do Trong et al. investigated a new SRS fiber array device (Nguyen Do Trong et al., 2011), shown in **Figure 5**, which consists of a halogen light source (AvaLight-DHc, Avantes, Netherlands), an illumination fiber and five detection fibers, a spectrometer, a CCD camera, data acquisition, and control equipment. Spectral data were collected at intervals of 0.15 mm between the detection fiber and the illumination fiber over a range of 0.3-1.2 mm. The setup could split the diffuse light into multiple wavelengths in the range of 500-1000 nm by means of a spectrometer and project them onto different areas of a CCD camera (S7031-1008S, Hamamatsu, Japan). Finally, a data acquisition card and a customized LabView program (LabView 8.5, National Instruments, USA) were used to collect spatially resolved information.

**Figure 6:**
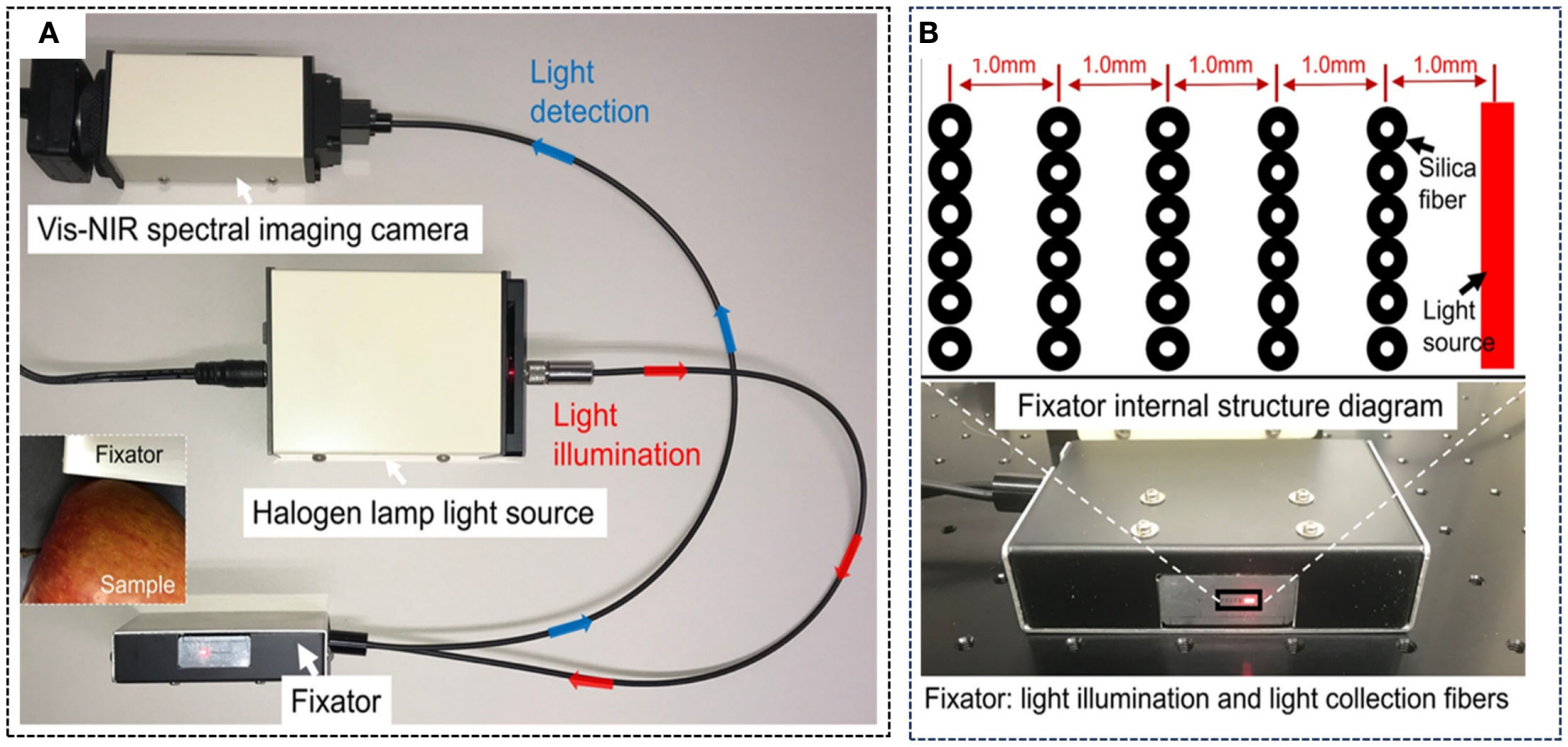
Vis-NIR SRS system (Ma et al., 2021b). **(A)** SRS detection systems. **(B)** Internal structure diagram of the fixator.

In order to collect spectral information at more distances, Ma et al. designed a Vis-NIR SRS system (Ma et al., 2021b), as shown in **Figure 6**, which consists of a 5 W halogen light source, a Vis-NIR HSI camera and 30 silica fibers (core diameter: 100 µm, cladding: 110 µm), with five groups of fibers, each consisting of six fibers, 1, 2, 3, 4 and 5 mm away from the light source, respectively. The 30 silica fibers installed in this SRS acquisition device including both horizontal and vertical spatial-spectral information of the sample under test, which could increase the exploration of the spatially resolved spectral information.

These fiber optic array-type devices are mainly arranged in the form of linear arrays (Nichols et al., 1997; Doornbos et al., 1999; Bogomolov et al., 2017) and circular arrays (Dam et al., 2001; Nguyen Do Trong et al., 2011; Bridger et al., 2021). Their arrangement can be designed according to the sample’s shape and structure’s size. Since the designed fiber array structure is fixed to detect the spectral information at once, it can save the measurement time as well as avoid the spectral error caused by the inaccuracy of the distance during the measurement. However, custom-designed fibers are more costly and require testing and calibration of the fiber arrays. The fiber optic array is only suitable for detecting samples with a flat surface for most agricultural products due to the irregularity of the sample detection fiber probe not well fitted to the sample surface. In addition, this system requires contact with the sample surface during the inspection process and is not friendly to liquids, easily polluted and vulnerable samples, so it needs to be continuously improved and developed.

### 2.3 CCD line scan type system

The fiber optic array detection method is suitable for measuring liquid samples because the integrated array probe can make good contact with the liquid surface. In addition, it is also well suitable for flat sample pieces, such as dried apples or tablets (Igne et al., 2015), etc., but it is easy to contaminate the sample with this contact detection method, so it needs to be cleaned frequently. In order to achieve a non-contact measurement method while detecting the spatially resolved information of the sample, researchers have developed a spatially resolved detection system based on the CCD line scan method.

**Figure 7:**
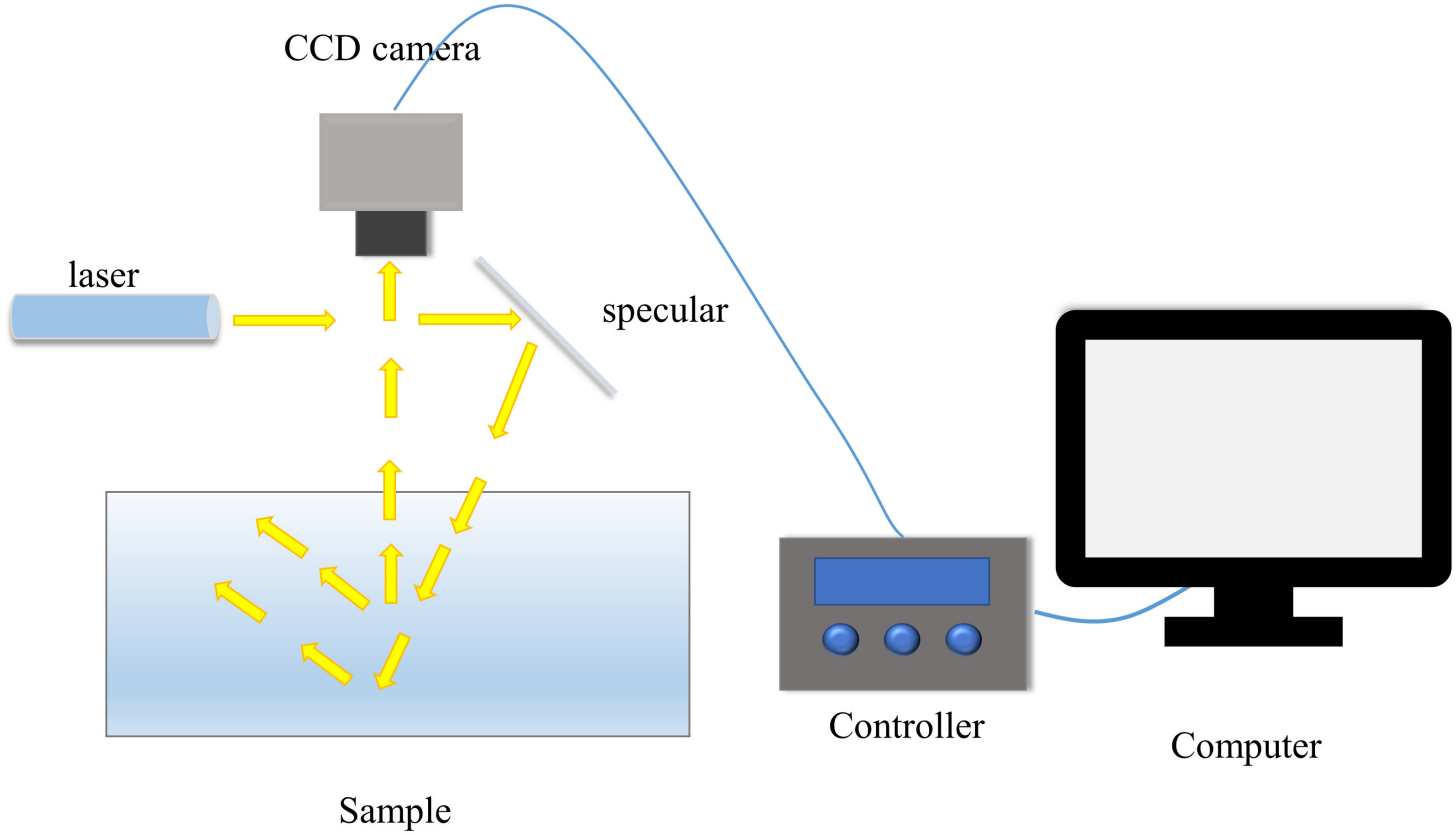
CCD line scan spatial resolution system (Kienle et al., 1996).

The spatially resolved system of CCD line-scan type is also called monochromatic imaging spatially resolved system, which is available for detecting the OP of a sample at a single wavelength. As shown in **Figure 7**, Kienle et al. (1996) used this approach for inspection. The system mainly consists of a laser diode as a light source, which is illuminated by a mirror at an angle of incidence of 5-10° on the object to be measured and detected by a CCD camera, and then the detected data are read out and processed by a computer.

**Figure 8:**
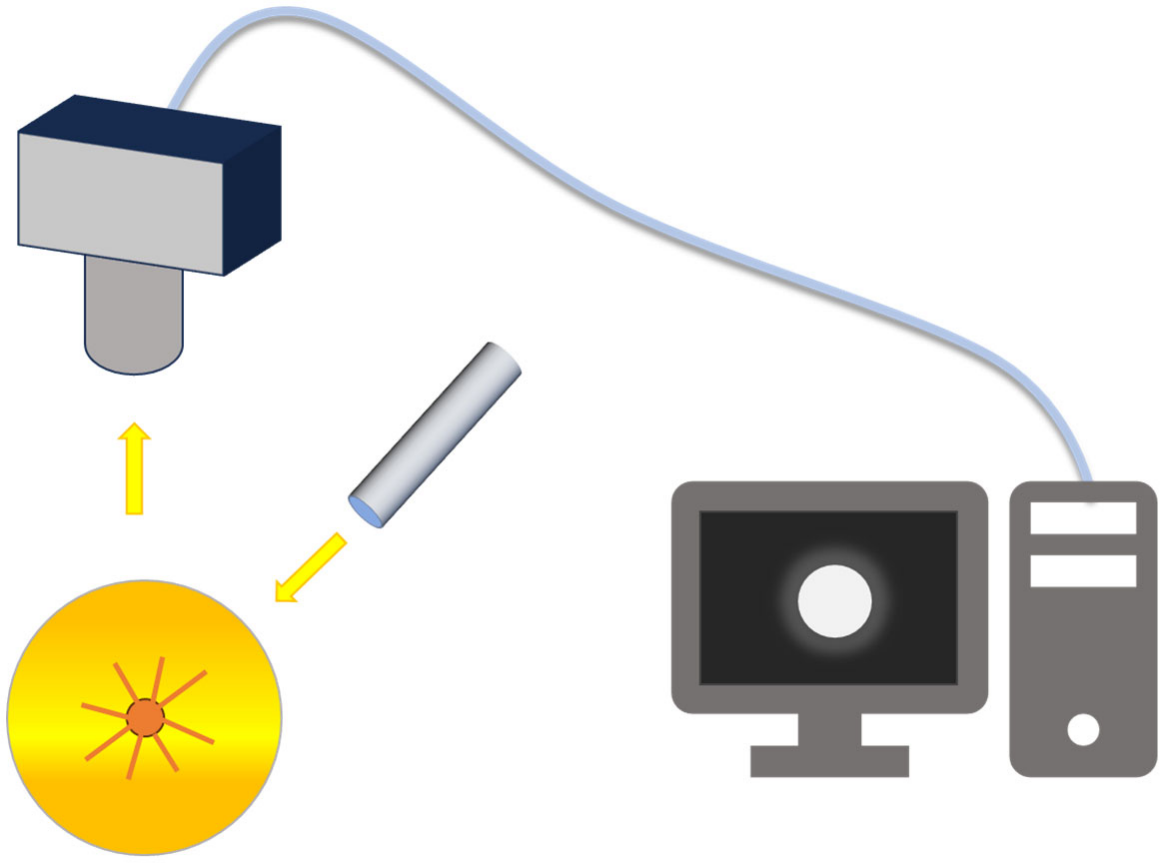
Laser diode-based optical properties device (Lorente et al., 2013).

Since laser diodes can only emit a single wavelength, this is not very friendly for analyzing multiple wavelengths. Therefore, researchers have pooled diodes at several different wavelengths for detection, which was used by Lorente et al. to detect the early ripeness of citrus fruits (Lorente et al., 2013). As shown in **Figure 8**, the system consists of a CCD camera, five solid-state laser diodes emitting at different wavelengths (532, 660, 785, 830, and 1060 nm), and a computer. In the acquisition process, the laser diodes are not integrated together for the acquisition, but the alternating form of replacing the diode of the corresponding wavelength each time to be used as a light source, so as to achieve the acquisition of spectral information at different wavelengths.

Conventional CCD imaging systems do not contain spectroscopic components inherently, and the acquired images are ordinary RGB images. Due to the theory of optics, only lasers or laser diodes can be used as light sources for CCD cameras. This limits the system to detecting the optical properties of the sample at a single wavelength. Although, at this stage, there is a way to detect spectral information in multiple wavelengths using diode module integration, it is still far from sufficient for analyzing continuous wavelengths. Moreover, the saturation of pixels occurs close to the light source point, so this area cannot be used for data analysis, and to avoid saturation, it is usually necessary to limit the exposure time (Kienle et al., 1996). This makes the CCD line-scan type system not well suited to the needs of the application, so a more optimized spatially resolved detection system is urgently needed.

### 2.4 Hyperspectral line-scan system

**Figure 9:**
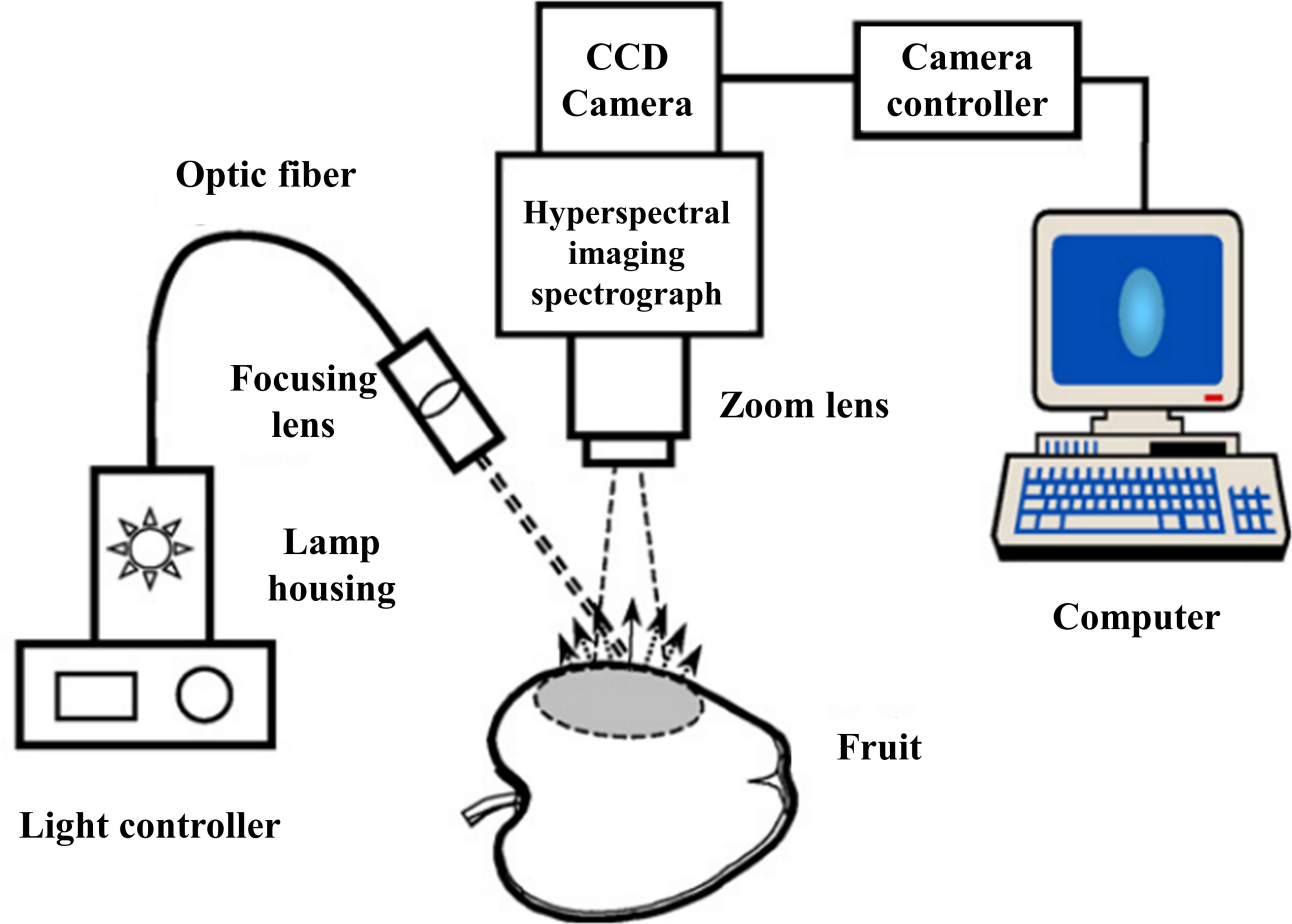
Hyperspectral imaging system for acquiring spectral scattering images (Peng and Lu, 2008).

For SRS, the more continuous wavelength bands the collected information contains, the more advantageous it is likely to be for subsequent data analysis and processing. In pursuit of acquiring spatially resolved spectral information in continuous bands in a non-contact system, researchers have combined hyperspectral imaging (HSI) techniques with SRS, and they have been widely developed and applied. As shown in **Figure 9**, Peng and Lu (Peng and Lu, 2008) used a spatially resolved line-scan system, which mainly consists of a back-illuminated camera (C4880-21, Hamamatsu Photonics, Hamamatsu Corp., Japan), a control unit, an imaging spectrometer (ImSpector V9, Spectral Imaging Ltd., Oulu, Finland), a quartz tungsten halogen lamp (Oriel Instruments, Stratford, CT, USA) and a circular open sample holder with a diameter of 30 mm. The light source is a 1.5 mm circular beam, and the hyperspectral imaging system line scan is 1.6 mm from the light source to avoid oversaturation of the CCD detector pixels.

**Figure 10:**
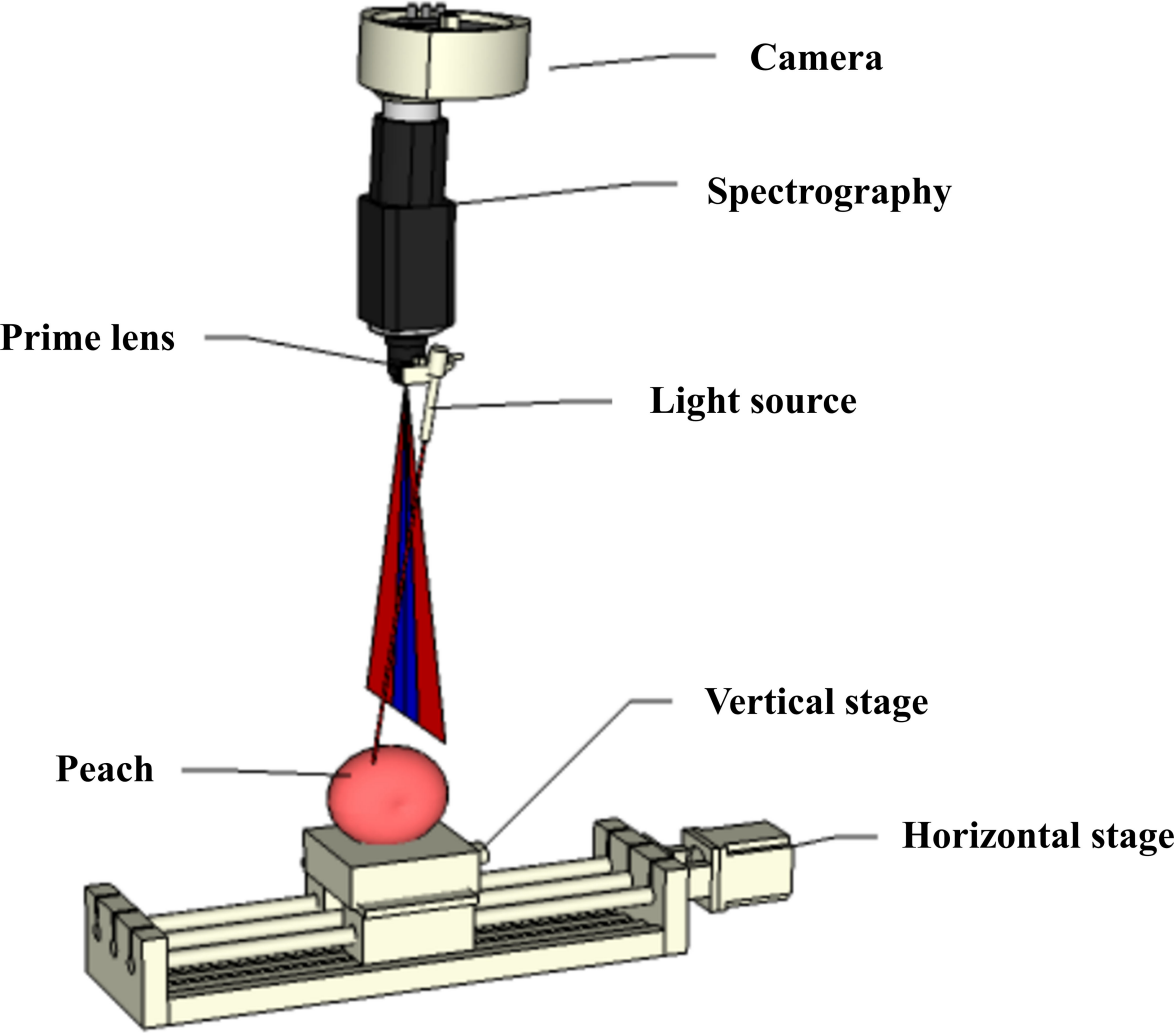
The Optical Property Analyzer (OPA) (Cen et al., 2011).

To make it easier to detect the OP of SRS, as shown in **Figure 10**. Cen and Lu (Cen et al., 2011) developed the Optical Property Analyzer (OPA), which consists of three main hardware components that are imaging, illumination, and sample positioning units. The imaging device mainly consists of an electron-multiplying CCD (EMCCD) camera (LucaEM R604, ANDORTM Technology, USA), an imaging spectrometer (ImSpector V10E, Spectral Imaging Ltd., Oulu, Finland), and a master lens (Xenoplan 1.9/35, Schneider Optics, Hauppauge, USA). An optical fiber connected to the focusing lens can be used to emit a point light source. The sample fixation device consists of a motorized horizontal stage (Twintrac, TSZ8020, US23T22104-8LS, US Automation, USA) with a maximum speed of 203 mm/s and a positioning accuracy of 0.0006 mm/mm, a vertically adjustable stage, and a holder for sample positioning. The integrated software program for OPA is developed in Microsoft Visual C#. It can control the light source, camera, and sample mounting platform for spectral and image data acquisition and also analyze and display the acquired information in real time to obtain the final scattering profile, absorption spectrum, reduced scattering coefficient, etc. Due to the powerful and convenient functions of this software, the workload of spectral data acquisition and analysis can be greatly reduced, and the efficiency of the sample acquisition and analysis can be improved.

**Figure 11:**
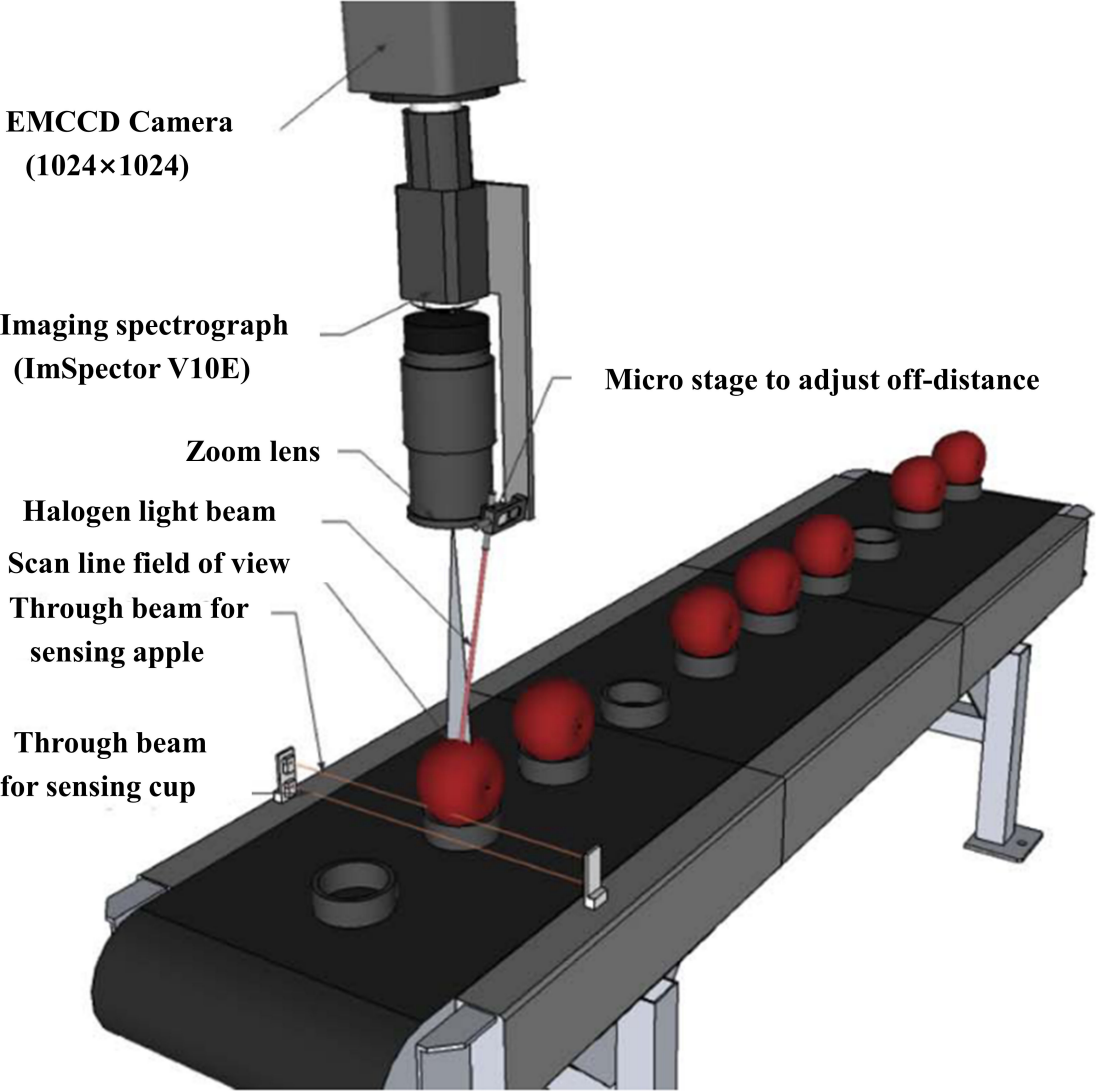
Online hyperspectral imaging system (OHIS) (Mendoza et al., 2011).

Mendoza et al. (2011) developed an online hyperspectral imaging system (OHIS) based on a hyperspectral line-scan type (**Figure 11**), which consists of a back-illuminated EMCCD camera, an imaging spectrometer (ImSpector V10E, Spectral Imaging Ltd., Oulu, Finland) covering a spectral region of 400-1000 nm. A near-infrared enhancement lens and a halogen light source (Oriel Instruments, USA). The computer is equipped with an image acquisition card and a camera acquisition program written in C++, through which the camera can be controlled for image acquisition. In order to capture the samples in real time and to increase the efficiency of the test, the device also uses a conveyor belt that can hold the samples. The imaging system of this OHIS operates at a rate of approximately one in two seconds. This system is the first to combine spatially resolved line sweep with online inspection. Although the system has good predictions, it is costly and still has some errors for curved samples.

While hyperspectral imaging inspection methods can realize the advantages of contactless, efficient, and high-resolution acquisition, they also have significant drawbacks. However, it is only suitable for detecting samples with flat surfaces or objects of considerable size, and if the surface curvature of the sample is large, the detected information will have a large error, so the detection device of SRS needs to be improved continuously.

### 2.5 Multi-channel hyperspectral imaging detection system

**Figure 12:**
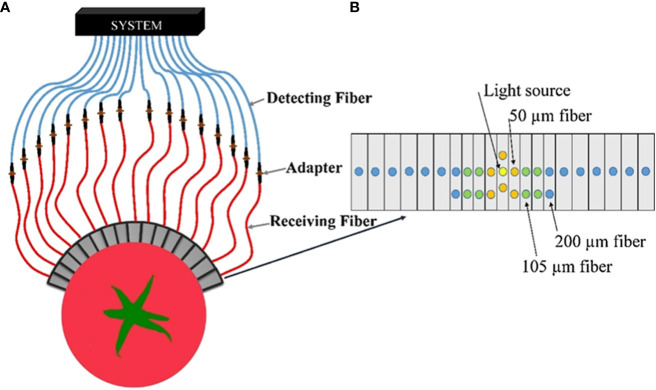
Multichannel hyperspectral imaging detection device (Huang et al., 2017). **(A)** Schematic of spatially resolved spectral acquisition. **(B)** Schematic of fiber arrangement.

The current device is only suitable for detecting samples with relatively flat surfaces, and the detection probes cannot fit closely for most agricultural products. When the detection sample is too large, the existing fiber array system makes it difficult to meet the requirements of the number of detection fibers and detection distance due to the limitations of the instrument. Although hyperspectral detection has excellent advantages, it has a narrow detection wavelength range and lacks flexibility for curved samples, which can cause significant errors. Therefore, Huang et al. (2017) designed a multichannel hyperspectral imaging detection device, as shown in **Figure 12**, which was based on a multichannel hyperspectral imager (Headwall Photonics, Inc., USA). The multichannel probe consists of a point source and 30 fibers of three sizes (i.e., 50 µm, 105 µm, and 200 µm). The light source fiber is connected to a 250 W halogen lamp, and the 30 fibers are permanently mounted on two sizes of aluminum cubes, giving the probe the flexibility to measure samples of different sizes and flat or curved surfaces at distances of 1.5-36 mm.

In general, the proposed SRS detection devices have their own advantages and disadvantages as well as applicable detection samples. The characteristics of these detectors are described in **Table 1**. Although the single fiber detection system is simple in structure, lowest cost, and flexible in collection, there will be a large measurement error and time-consuming, so it is not suitable for a large number of sample collections. Fiber array type system can achieve once-time acquisition at different distances to improve detection efficiency and accuracy, but the cost is higher than single-fiber detection systems with the need to detect samples as smoothly as possible, and contact measurements are prone to sample contamination, so the scope of use is also very limited. CCD line scan type system can realize non-contact measurements but cannot collect SRS in the continuous wavelength band. The hyperspectral line-scan system can acquire spectral information in continuous wavelength bands and are well suited for the detection of a large number of samples, while they are less friendly to the detection of samples with curvature, such as apples, peaches, oranges, etc. Multi-channel hyperspectral imaging detection system can detect the spectra of some curvature samples, but the cost is the highest, and there is still a large error for some samples with large curvature. In general, although these systems have detected the spatially resolved information of samples to a great extent, they are still not friendly enough for the detection of irregular objects because of their detection limitations, so there is still a huge space that could be developed and innovated for the detection systems of SRS.

**Table 1:** Summary of studies on the types of detection devices for agricultural products.

Detection systems	Objects	Wavelength(nm)	Detection distance(mm)	Characteristics	References
**SF**	Beef	490-950	Incident fiber Left: 9.0-6.5 Right: 4.0-7.0	Detection flexibility allows the selection of the optimal distance	(Xia et al., 2007)
	Apple	190-1070	2, 4, 6, 8, 10, 12, 14, 16	Easy operation, reduce error	(Ye et al., 2021)
	Pear	500-1000	-0.15, -0.1, -0.05, 0, 0.05, 0.1, 0.15	–	(Hu et al., 2017)
	Onion	710-950	–	The laser system has a slightly better optimal single point ratio than the NIRS system	(Sun J. et al., 2020)
	Rabbit	350-1000	5, 10, 15	Detecting distance slidable	(Yuan et al., 2022)
**FA**	Milk	550-1690	1-2.5	Optimal combination of minimum fiber counts	(Watte et al., 2016)
	Milk	400-995	0.28-1.96	Full-spectrum analysis replaced by two wavelength-specific sensor measurements	(Bogomolov et al., 2017)
	Pork	600-1100	6, 9, 12, 15	Improve detection efficiency	(Wen et al., 2010; Zhang et al., 2010)
	Pork	600-1100	6, 9, 12, 15	Efficient, low cost	(Wang J. et al., 2017)
	Apple	500-1000	0.3-1.2	Efficient	(Nguyen Do Trong et al., 2014a; Nguyen Do Trong et al., 2014b)
	Apple	600-1100	1, 2, 3, 4, 5	Portable, high efficiency	(Ma et al., 2021b)
	Kiwifruit	660-1000	1, 2, 3, 4, 5	Portable, high efficiency	(Ma et al., 2022)
	Wood	600-1100	2, 3, 4, 5	Portable, high efficiency	(Ma et al., 2021c)
	Cattle	500-900	0.5, 1.0, 1.5, 2.0, 2.5	Fiber integration, high efficiency	(Palendeng et al., 2020)
**CL**	Milk	800-1065	–	Fast, portable and inexpensive	(Kalinin et al., 2013)
	Apple	650-980	–	Detecting distance slidable	(Mollazade and Arefi, 2017)
	Banana	532, 660, 785, 830, 1060	–	Specific wavelength, non-contact	(Adebayo et al., 2016)
	Citrus	532, 660, 785, 830, 1060	–	Specific wavelength, non-contact	(Lorente et al., 2013)
	Wood	808	Dry: Parallel: 20 Perpendicular: 10 Wet: Parallel: 25 Perpendicular: 15	Non-contact	(Kienle et al., 2008)
**HL**	Milk	530-900	1.6-20	Non-contact, easy operation	(Qin and Lu, 2007)
	Apple	500-1000	1.6-9	Easy to operate	(Qin et al., 2007; Qin et al., 2009; Lu et al., 2010)
	Apple	450-1000	–	Easy to operate	(Peng and Lu, 2008)
	Apple	600-1000	–	Easy to operate	(Huang and Lu, 2010)
	Apple	500-1000	0-9	Easy to operate, with analysis software	(Cen et al., 2012b; Cen et al., 2013)
	Apple	450-1050 (Scatter) 460-1100 (Vis/SWNIR)	–	Realized hyperspectral online detection	(Mendoza et al., 2014)
	Apple	500-1000	20	Easy to operate, with analysis software	(Zhu et al., 2016)
	Peach	550-1650	1-9	Easy to operate, with analysis software	(Cen et al., 2011; Cen et al., 2012a)
	Peach	550-1000	1-9	Easy to operate, with analysis software	(Sun Y. et al., 2020; Sun Y. et al., 2021)
	Cucumber	700-1000	37-55	Easy to operate, with analysis software	(Lu et al., 2011)
	Tomato	500-950	0-10	Easy to operate, with analysis software	(Zhu et al., 2015)
	Wood	1000-1600	1, 3, 5 (Thicknesses)	Non-contact methods, push-broom manner	(Ma et al., 2018; Ma et al., 2019b)
	Wood	1002-2130	–	Non-contact methods, push-broom manner	(Ma et al., 2019a)
	Tea	967.11-1700	–	Non-contact methods, push-broom manner	(Mishra et al., 2019)
**MHI**	Apple	550-1650	1.5-36	Detects longer distances and higher accuracy	(Huang et al., 2020b)
	Peach	550-1650	1.5-36	Detects longer distances and higher accuracy	(Huang et al., 2022)
	Tomato	550-1300	1.5-36	Detects longer distances and higher accuracy	(Huang et al., 2018b) (Huang and Chen, 2018)
	Tomato	550-1650	1.5-36	Detects longer distances and higher accuracy	(Huang et al., 2018c; Huang et al., 2020a)

SF, Single fiber system; FA, Fiber array type system; CL, CCD line-scan type system; HL, Hyperspectral line-scan system; MHI, Multi-channel hyperspectral imaging detection system.

## 3 Development of spatially resolved spectral analysis methods

SRS collects spectral information at different distances and then needs to be processed. In most cases, the optical properties are obtained based on SRS techniques, and then the association between sample properties and optical properties is analyzed on the basis of absorption coefficients and reduced scattering coefficients. Direct analysis is also used to detect the properties by processing the spectral information at different distances. At this stage, researchers have done a lot of work on the basis of optical properties and direct analysis, which provides powerful and effective support for the development of SRS in the future.

### 3.1 Optical properties methods

Among the methods of measuring OP in biological tissues, there are currently two ways: direct and indirect measurement, respectively. In the direct measurement method, Beer-Lambert’s Law is used to calculate the OP within the tissue. In this method, the optical properties of the tissue are calculated by measuring parameters, such as complete attenuation transmission and collimated transmission of a slice sample. Although the direct analysis calculation method is simpler, its detection process is more complex, requiring slicing and strict requirements for the thickness of the slice (Cheong et al., 1990). The indirect measurement method is mainly used to solve the OP by inversion. Generally, researchers classify indirect measurements into non-iterative and iterative approaches according to whether the inversion process includes a parameter iteration step. The non-iterative approach can be used to solve the optical properties of the optical transmission model directly from the measured values. One of the more commonly used methods is the Kubelka-Munk method (Kubelka, 1948), but the accuracy of its measurements is not high, requiring assumptions on various conditions. The iterative approach is to evaluate the OP by inverting the parametric equations for several iterations so that the measured values are within the specified error range. Although this method is more complicated, the measured optical properties are more accurate than other methods. Spatially resolved techniques are also usually applied by using indirect iterations to find the OP within the sample tissue.

For the transmission of light in biological tissues, a series of complex optical phenomena occurred, such as absorption, scattering, reflection, refraction, interference, and diffraction of light. Although Maxwell’s set of equations based on electromagnetic theory can describe the light propagation process in tissues mathematically (Yang et al., 2021; Katsumata, 2022), the equations cannot be solved directly due to the complexity of biological tissues. In order to study only the particle properties, such as absorption and scattering of light, the fluctuating properties of light, such as interference, diffraction, and polarization, can be ignored. Researchers have proposed the Radiative Transfer Equation (RTE), which is more accurate in describing the transport properties of light in tissues (Martelli et al., 2021; Hank et al., 2023), but the model is still complex and has many parameters. Therefore, researchers usually have used simplified transport model methods as well as numerical methods to solve the optical properties (Lu et al., 2020). The commonly used transport models are the diffusion approximation and the P3 approximation, which is the theoretical model to describe the spatially resolved diffuse reflection near the light source. The P3 approximation model is a third-order form of the radiative transfer model. Since the P3 approximation is more accurate, it can be used in place of the diffusion approximation (Wang, 2020; Wang, 2022). In addition, numerical methods include Monte Carlo (MC) simulation (Chong and Pramanik, 2023; Colas et al., 2023; Lan et al., 2023), Adding-Doubling model (Xie and Guo, 2020; Sun et al., 2022) and finite element methods (Morimoto et al., 2020).

The diffuse approximation equation is a simplified form of the radiative transfer equation, which has the ability to be simplified by satisfying two assumptions. 1) The medium is a strongly scattering medium, i.e. μs'≫μa . 2) The SDD is greater than the mfp’ (mean free path), i.e. *r*>mfp’. In addition, the incident light scattering step in the tissue is considered to be isotropic radiation. The diffuse approximation equation can be expressed as:


$$\frac{\partial \Phi (\vec{r},t)}{c\partial t}+\mu_{a}\Phi (\vec{r},t)-\nabla \cdot [D\nabla \Phi (\vec{r},t)]=S(\vec{r},t)$$

where *c* is the spreading speed of light through the medium, is the radiation fluence rate, $\vec{r}=(x,y,z)$ is a point within the medium, $D={\left[3(\mu_{a}+\mu_{s}^{'})\right]}^{-1}$ is the diffusion coefficient, $S(\vec{r},t)$ is each homogeneous light source. This equation can be used to describe the transmission of light through some objects with geometric shapes, such as semi-infinite, flat, cylindrical, spherical, etc. (Farrell et al., 1992; Kienle et al., 1998), which provides a good application for detection of OP of most samples. Depending on different illumination methods such as steady-state point illumination, pulsed point illumination, frequency-modulated point illumination, and spatially modulated area illumination, OP techniques have also evolved into spatially resolved techniques, time-resolved techniques, frequency domain resolved techniques, and spatial frequency domain techniques (Lu et al., 2020).

Based on the theory of diffuse approximation equations, Farrell et al. proposed a diffusion-theoretic model for SR steady-state diffuse reflection in the study of nondestructive determination of OP in humans (Farrell et al., 1992). The model can be used to describe the directional dependence of light diffuse reflection in biological tissues when irradiated by an infinitesimal amount of light. By comparing the predictions of the model with MC simulations and with tissue simulation models, it was found that the model can accurately describe the reflectance at radial distances as small as 0.5 mm. Thus, the model can provide an effective method and basis for later researchers to calculate and separate the OP. In this model, the diffuse reflection of the medium is computed as a boundary flow from a single isotropic point source located at the mfp’ depth of the medium transport. The model is applied to surfaces with matched or unmatched refractive indices. The equations of this diffusion model are as follows:


$$R\left(r\right)=\frac{a^{\prime }}{4\pi }[\frac{1}{\mu_{t}}(\mu_{eff}+\frac{1}{r_{1}})\frac{\exp(-\mu_{eff}r_{1})}{r_{1}^{2}}+(\frac{1}{\mu_{t}}+\frac{4A}{3\mu_{t}^{'}})(\mu_{eff}+\frac{1}{r_{2}})\frac{\exp(-\mu_{eff}r_{2})}{r_{2}^{2}}]$$

where *r* is the source-detector distance, $r_{1}={\left(z_{0}^{2}+r\right)}^{1/2}$ is the real distance from the detector to the light source, $r_{2}={\left({\left(z_{0}+2z_{b}\right)}^{2}+r^{2}\right)}^{1/2}$ is the length from the mirror light source to the detector. $\mu_{eff}^{'}={\left[3\mu_{a}(u_{a}+\mu_{s}^{'})\right]}^{1/2}$ is the effective reduction factor, $a^{\prime }=\mu_{s}^{'}/(\mu_{a}+\mu_{s}^{'})$ is an albedo of transmission, $\mu_{t}^{'}=\mu_{a}+\mu_{s}^{"}$ , is the overall decays value, $z_{0}={\left(\mu_{a}+\mu_{s}^{'}\right)}^{-1}$ is the mfp’, $z_{b}=2AD$ , *A* is the object’s internal reflection coefficient, *A*=1 when the tissue and surrounding media boundaries match, and *A*=0.2190 when the relative refractive indices of the tissues *n*=1.35. While refractive index is known to be wavelength dependent, most reports assume that the *n* is constant, an assumption that is subject to potential uncertainty, such as for many fruits and foods *n*=1.35.

Later, Kienle and Patterson (1997) introduced radiant energy flow rate following Haskell et al. (1994). The diffuse reflectance is expressed through the brilliant energy flow rate and luminous flux, which better minimizes errors and thus more precisely characterizes the transmission of light in biological tissues. This equation can be expressed as:


$$\Phi \left(r,z=0\right)=\frac{1}{4\pi D}[\frac{\exp(-\mu_{eff}r_{1})}{r_{1}}-\frac{\exp(-\mu_{eff}r_{2})}{r_{2}}]$$

The diffusive approximation equation can be organized as follows:


$$\begin{array}{c} R(r)=\frac{C_{1}}{4\pi D}[\frac{exp(-\mu_{eff}r_{1})}{r_{1}}-\frac{exp(-\mu_{eff}r_{2})}{r_{2}}]+\\ \frac{C_{2}}{4\pi }[\frac{1}{\mu_{t}^{'}}(\mu_{eff}+\frac{1}{r_{1}})\frac{exp(-\mu_{eff}r_{1})}{r_{1}^{2}}+(\frac{1}{\mu_{t}^{'}}+2z_{b})(\mu_{eff}+\frac{1}{r_{2}})\frac{exp(-\mu_{eff}r_{2})}{r_{2}^{2}}] \end{array}$$

where $C_{1}=\frac{1}{4\pi }\int_{2\pi }\left[1-R_{\text{fres }}\left(\theta \right)\text{cos}\theta d\omega \right]$ and $C_{2}=\frac{3}{4\pi }\int_{2\pi }\left[1-R_{\text{fres }}\left(\theta \right){\text{cos}}^{2}\theta d\omega \right]$ are coefficients generated by the refractive index of the medium and $R_{\text{fres }}(\theta )$ is the Fresnel coefficient. When rate of refraction *n*=1.35, C1 and C2 are 0.1277 and 0.3269, respectively (Cen and Lu, 2010). This solution is considered to be more accurate in describing the light propagation process, therefore it is widely used (Cen et al., 2010).

At present, these two are the most commonly used mathematical fitting models for spatially resolved spectra, and their analytical solutions are obtained under extrapolation boundary conditions (EBC). The source is assumed to be each homogeneous radiation source at one mfp’ below the sample surface. Therefore, the precision of OP parameter inversion is not only related to the precision of instrumental measurements but also depends on the precision of parameter inversion algorithms. Cen and Lu further optimized the curve fitting algorithm by using the nonlinear least squares method as the Trust-region-reflective least squares method, and the raw data were logarithmically and integrally transformed and relatively weighted before fitting to improve the OP predictions (Cen et al., 2010). The prediction of the OP was enhanced by using logarithmic and integral transformations of the original data and relative weighting before fitting.

However, many factors affect the accuracy and error in the acquisition and processing of the spectra and the inversion of the parameter equations. Usually, normalization is required before curve fitting. However, the standard normalization method directly divides the first value of the spatially resolved diffuse reflectance spectrum, which contains considerable noise and acquisition errors. It has a great impact on the inversion of the later parametric equations. The diffuse approximation equation is invalid when it is close to the light source, i.e. (*r*<1 mfp’), and the reflection signal is weaker, and the signal-to-noise ratio (SNR) is lower when the acquisition is farther away, which is not conducive to the inversion of the optical parameters. Therefore, an effective interval selection for the acquired spatially resolved spectra is also needed before curve fitting. Farrell et al. suggested that the SDD should be greater than one mfp’ (Farrell et al., 1992), and Nichols et al. recommended minimum and maximum distances of SDD are 0.75-1 mfp’ and 10-20 mfp’, respectively (Nichols et al., 1997). Nevertheless, for most of the unknown samples with unknown OP, it is impossible to calculate the mfp’ directly. So Wang and Lu et al. proposed a mean normalization method to optimize the normalization along with a method to optimize the diffuse reflectance spectral interval for the inversion of OP based on the relative error contour (Wang A. et al., 2017).

The inversion of the parametric equations is performed by fitting the diffuse reflectance spectral data to an analytical solution of the diffuse reflectance approximation equations to calculate the absorption coefficients and the reduced scattering coefficients. Cen and Lu used the spectral SNR to optimize the endpoint of the spectrum (Cen and Lu, 2010), but the starting point of the spectrum is fixed at 1.5 mm for systematic reasons, which still leads to large measurement errors for samples with mfp’ greater than 1.5 mm measurement error. Therefore, to further solve the problems of fitting, Wang and Lu proposed the step-by-step parameter inversion method (Wang A. et al., 2017), which is based on the OP and the mfp’ obtained by the one-step fitting method, and then re-determine the better spectral interval based on the optimized starting point and end point before the second fitting to obtain the better OP. The method is effective in improving the optical parameters. This method can significantly improve the inversion accuracy of optical parameters.

However, in the process of inversion, the traditional inversion algorithm does not meet the requirements due to the single-layer and double-layer tissues of the sample under test. The traditional inversion algorithm is to equate the outer tissue of the sample with the inner tissue as a layer. For samples with a thin outer skin, the effect of having a thickness less than the mfp’ will not be significant, but for pieces with a thicker outer skin, the effect will be more meaningful if they are equated to a monolayer of tissue. There are already diffuse reflection equations for light transmission in single- and double-layer tissue that can be used as an approximate solution (Kienle et al., 1998; Cen and Lu, 2009), but the light transmission in double-layer tissue is a more complex transmission model involves more parameters, the accuracy of the fitted parameters will be increasingly poor. Accordingly, the inversion of parametric equations for multilayer tissues still needs to be continuously explored and studied by researchers.

The most commonly used method in numerical calculation methods is MC, which is a statistical method with random sampling and has been widely used to simulate the propagation of light (Tarasov et al., 2021; Sassaroli et al., 2022). This method can simulate the light transmission process by tracking the trajectory of a massive photon through the tissue and finally calculate the optical parameters we need. The advantages of MC are low cost, high accuracy, and high flexibility. However, it also has obvious drawbacks, which are computationally intensive, time-consuming, and not conducive to rapid detection, so the method is often used to test the accuracy of other calculation methods. When the MC method simulates the light transmission law in tissues, it mainly simulates the particle properties of light, i.e., the absorption and scattering of light and other properties. Its typical simulation specifically includes the processes of photon generation, initialization, migration, absorption, scattering, boundary condition processing, and extinction judgment (Wang et al., 1995). Currently, the program developed by Wang and Jacques (1992) based on C language can be used for multilayer organization, which consists of two subroutines, Monte Carlo Multi-Layered (MCML) and Convolution (CONV), where MCML is used to simulate the transmission of light beams in the organization. CONV is used to convolve the simulated data of MCML and output the results. Based on the disadvantages of MC time consumption, Hu et al. (2020b) optimized it and accelerated its simulation. Sun et al. used the Monte Carlo multilayer (MCML) technique to simulate the propagation of light through the fruit by comparing it with the diffuse reflection curve, thus confirming the accuracy of the MC simulation of the OP (Sun C. et al., 2021).

Although MC is usually used as a reference method and is more accurate, it needs a huge number of photons to be simulated at a time, which is computationally intensive and cannot meet the rapid detection of OP of biological tissues despite the fact that its speed has been improved. The finite element method (FEM) is also one of the commonly used numerical methods, which is more flexible and fast based on accuracy (Vasudevan and Narayanan Unni, 2021). Lee et al. (2004) used the FEM method to study the propagation of light in a double-layer medium and found that the accuracy of the method and MC were almost the same by comparison. Wang et al. (2016) investigated the optimal computational results of finite elements under three boundary conditions and demonstrated that the finite element approach can be used to improve the measurement of OP for spatially resolved techniques. Whereas, at this stage, there are few analytical methods using finite elements in the study of calculating the OP of spatially resolved spectra, which is a promising method for the numerical calculation of OP.

### 3.2 Direct analysis methods

While analysis methods based on OP can find absorption coefficients and approximate scattering coefficients more accurately, they are labor-intensive, algorithmically complex, and have large accuracy errors. In order to directly and accurately analyze spatially resolved (SR) spectra as well as to simplify the analysis steps, the researchers proposed a direct analysis method.

Huang and Chen (2018) proposed an analytical method of spectral combination when employing a multichannel SRS system to detect tomatoes by creating a Partial Least Squares Discriminant Analysis (PLSDA) model of each of the 15 single SR spectra combinations to determine the best single SR combination for classification. Next, the best SR combination was combined with the remaining 14 SR combinations to select the best two-spectrum combination, then the best spectrum was combined with the remaining 13 single SR combinations to create the best tri-spectral combination, and so on until the accuracy of the combined SRS for classification is not further improved.

As for quality detection of peach, Huang et al. (2022) proposed a spectral difference technique to deal with spatially resolved spectral information. The method initially collects 30 relative spatial spectra for each sample at different SDD scales, which are calculated as follows:


$$R(i)=\frac{I_{s}(i)-D_{s}(i)}{I_{r}(i)-D_{r}(i)}$$

where *R* is the relative spectrum, *I* is the spectral information of the sample, *D* is the blackboard, *i* is for single fiber, *i* = 1, 2, 3,…, 30, and the subscripts *r* and *s* represent the white Teflon and the sample, respectively. Since the device has detection fibers arranged symmetrically, each symmetrical pair of SR spectra is averaged over the same SDD, resulting in 15 SR spectra whose distances range from 1.5-36 mm. The difference spectrum is obtained by differencing the spatially resolved spectrum of the first position (SR1) with the spatially resolved spectra of the other SDDs with the following calculation equation:


D(i)=R(i)−R(1), i=2,3,4,5,⋯,15

where *R*(*1*) is the SR spectrum at the first SDD of 1.5 mm, and *D* is the subtraction spectrum, after that, it is referred to as the differential reflectance (DR) spectrum, and the final 14 DR spectra contain different spatial resolution information compared to the SRS.

Ma et al. (2021b) proposed a method for reference-free reflectance calculation in assessing apple quality by averaging the light centers at distances of *d* mm and *d*+Δ mm for the diffuse reflected light intensity (*i*) collected by the optical fibers and named *i_d_* and *i_d_*_+Δ_, respectively. When the intensity of the spatially resolved spectrum is *i_ref_*, the spectral difference between fibers at different distances can be expressed as:


$$A_{diff}=-log_{10}(\frac{i_{d+\Delta }}{i_{ref}})-(-log_{10}(\frac{i_{d}}{i_{ref}}))$$

where *A_diff_* is the difference in absorption spectra. The equation can be simplified as:


$$A_{diff}=-log_{10}(\frac{i_{d+\Delta }}{i_{d}})$$

In this way, the calculation formula of the spectrum can be simplified, and the black-and-white correction of the spectrum can be canceled, which makes the spectral inspection more efficient and convenient. The ratio of diffuse light intensity (*R_Ratio_*) is calculated as follows:


$$R_{\text{Ratio }}=\frac{i_{d+\Delta }}{i_{d}}$$

Finally, smoothing of the spectral data using Savitzky-Golay filters enables the spectra to achieve better results in modeling analysis.

The direct analysis method of SRS simplifies the analysis steps, and although it is not more accurate than the OP method, it has the same good prediction effect for the quality detection of agricultural products. There are few direct analysis methods used so far. If a better direct analysis method can be proposed to predict the quality of products, not only the analysis method is simple and fast, but also the quality prediction accuracy is more accurate, then the detection efficiency of SRS will be significantly improved.

## 4 Application of spatially resolved spectroscopy in agricultural products

Although SRS has been widely adopted in the biomedical field, its application in the agricultural field is still relatively limited. At present, the application of spatially resolved technology is mainly concentrated in the field of edible agricultural products, such as meat, dairy, fruits, and vegetables, and less application in other areas, such as forestry, animal husbandry, etc. The technology of detecting the quality or classification of agricultural products by SRS is more mature. In the subsequent sections, the latest research and specific applications of SRS in agriculture were presented and summarized in detail.

### 4.1 Applications of dairy field

In the field of dairy products, SRS is more widely used in the detection of milk. Because milk is rich in nutrients such as protein, fat, vitamins, and minerals, it is very easy to be absorbed by the human body, so it is very popular among human beings. To ensure the quality of raw milk or to prevent adulteration during the sale process, it is necessary to test the quality of raw milk. Watte et al. developed a global optimizer that can calculate the optimal configuration of fibers, by which the number of detected fibers can be minimized while maintaining the validity of the OP evaluation, making the detection optimal with cost savings. The design achieved good results for the evaluation of the OP of milk, with a root mean square error of the prediction (RMSEP) of 0.382 cm^-1^ and R^2^ = 0.996 for the reduced scattering coefficient values (Watte et al., 2016). Kalinin et al. used a dual-channel short-wave near-infrared spectrometer as a detection device. The results showed that the RMSEP of proteins using a combination of scattering and transmission spectroscopy could reach 0.25% wt. (Kalinin et al., 2013). Bogomolov et al. developed and utilized a fiber-optic array-based detection device with eight channels of probes to analyze the quality of milk and improve the accuracy of fat and protein detection, The root mean square errors (RMSE) for the different validation methods were less than 0.10% for fat content and less than 0.08% for total protein content, respectively. (Bogomolov et al., 2017). The optimal sensor configuration was proposed to replace the full spectrum analysis with LED in specific wavelength bands, which provided a faster and more mature application for milk detection. Qin and Lu used a hyperspectral line-scan detection device to analyze the fat content in milk. They found that the absorption coefficient and the reduced scattering coefficient at 600 nm were closely correlated with the fat content of milk, while the R^2^ were 0.995 and 0.998, respectively, which verified the feasibility of HSI in detecting the milk content (Qin and Lu, 2007).

As shown in **Tables 1** and **2** in dairy product testing, researchers have used different spatially resolved detection systems to detect milk’s fat and protein content to achieve good prediction results. However, more milk is currently detected, and the approach will definitely be developed toward a broader range of dairy products in future applications.

**Table 2:** Summary of studies about the quality detection of agricultural products.

Products	Species	Applications	Methods	Accuracy	References
**Dairy**	Milk	Fat and protein contents	PLS	RMSEP_f_ ≤ 0.08% RMSEP_p_=0.21%	(Kalinin et al., 2013)
	Milk	Fat and protein contents	GA	µ_a_:R^2^ = 0.965 µ_s_’:R^2^ = 0.996	(Watte et al., 2016)
	Milk	Fat and protein contents	PLSR, JVSPO	RMSEP_f_<0.10% RMSEP_p_<0.08%	(Bogomolov et al., 2017)
	Milk	Fat content	PLS	µ_a_: R^2^ = 0.995 µ_s_’: R^2^ = 0.998	(Qin and Lu, 2007)
**Meat**	Beef	Tenderness	–	p<0.0001, R^2^ = 0.59	(Xia et al., 2007)
	Pork	Tenderness	–	µ_s_’: R^2^ = 0.8349	(Zhang et al., 2010)
	Pork	myoglobin	–	R^2^ = 0.955	(Wen et al., 2010)
	Pork	moisture content	SPA, PLSR	R^2^ = 0.8078	(Wang J. et al., 2017)
**Fruit**	Apple	Firmness and SSC	MLR, LCV	F: r=0.88, SEP=5.66N SSC: r=0.82, SEP=0.75%	(Qin et al., 2007)
	Apple	Firmness and SSC	MLR, MLD	Firmness: R=0.894, SEP=6.14 N; SSC: R=0.883, SEP=0.73%	(Peng and Lu, 2008)
	Apple	Firmness and SSC	MLR, LCV	Firmness: R=0.844 SSC: R=0.864	(Qin et al., 2009)
	Apple	Bruise detection	–	–	(Lu et al., 2010)
	Apple	Mealiness	PLS-DA	Accuracy>93%	(Huang and Lu, 2010)
	Apple	Firmness and SSC	PLSR	F: r_GD_=0.892, r_RD_=0.863 SSC: r_GD_=0.892, r_RD_=0.863	(Cen et al., 2012b)
	Apple	Mechanical and structural properties	ANOVA, LSD	Acoustic/impact firmness GD: r=0.870–0.948 GS: r=0.334–0.993 Young’s modulus GD:r=0.585–0.947 GS: r=0.292–0.694	(Cen et al., 2013)
	Apple	Quality grades: firmness, SSC	LDA	Scattering technique Firmness: 77.9%-98.2% SSC: 62.0%-91.7% Vis/SWNIR technique Firmness: 87.3-97.6% SSC: 77.10-92.3%	(Mendoza et al., 2014)
	Apple	Microstructure, textural quality	–	–	(Nguyen Do Trong et al., 2014b)
	Apple	Firmness and SSC	PLS	Firmness: R^2^ = 0.71, RMSEP=9.68N SSC: R^2^ = 0.81, RMSEP=0.69%	(Nguyen Do Trong et al., 2014a)
	Apple	Bruise detection	PLS	Rp=0.848-0.919, RMSEP=32.4-50.7	(Zhu et al., 2016)
	Apple	Mealiness classification	PCR, PLSR, ANN	Non-mealy: 76% Mealt: 82% Fresh: 88% Semi-mealy: 59%	(Mollazade and Arefi, 2017)
	Apple	Varieties	PLSDA	Classification accuracies=0.994,	(Huang et al., 2020b)
	Apple	Firmness and SSC	CARS, PLSR	Firmness: R^2^ = 0.96, RMSE_cal_=0.37N, SSC: R^2^ = 0.87, RMSE_cal_=0.71N	(Ma et al., 2021b)
	Apple	Anthocyanins	PLS	Skin: R^2^>0.95, Whole flesh R^2^ = 0.74	(Ye et al., 2021)
	Peach	Maturity/quality assessment	PLS, PCA, LS-SVM	Firmness: 0.794, SSC: 0.504, Skin lightness: 0.898, Flesh lightness: 0.741	(Cen et al., 2011; Cen et al., 2012a)
	Peach	Tissue structural and biochemical properties	SPA, PCA	Membrane permeability µ_s_’=-0.962-0.743	(Sun Y. et al., 2020)
	Peach	Bruise detection	ANOVA, LSD SVM, PLSDA, C-SVC	µ_a_=76.25%, μ_s_’=76.25%, µ_a_×μ_s_’=84.75%, µ_eff_=84.5%	(Sun Y. et al., 2021)
	Peach	Firmness and SSC	PLS	Firmness: 0.853, SSC: 0.839	(Huang et al., 2022)
	Peach	pear porosity	ANOVA	760nm: R^2^ = 0.66 835nm: R^2^ = 0.57	(Joseph et al., 2023)
	Kiwifruit	Firmness, SSC, pH	PLSR	Firmness: R^2^ = 0.37, SSC: R^2^ = 0.81, pH: R^2^ = 0.59	(Ma et al., 2022)
	Pear	Optical property analysis (µ_a_, μ_s_′)	–	µ_a_=0.10-0.61cm^-1^ µ_s_’=12.5-9.5cm^-1^	(Hu et al., 2017)
	Banana	Chlorophyll, elasticity, SSC, ripeness	ANN	CH: R=0.9768-0.9807, EL: R=0.9553-0.9759, SSC: R=0.9640-0.9801, RI: classification, accuracy=97.53%	(Adebayo et al., 2016)
	Citrus	Early decay detection	GL, LDA	Classification accuracy=96.1%	(Lorente et al., 2013)
**Vegetables**	Cucumber	Defect detection	–	–	(Lu et al., 2011)
	Onion	Detecting internal rots	PLSDA	–	(Sun J. et al., 2020)
	Tomato	Maturity classification	PLSDA, SVMDA	Classification accuracy=81.3–96.3%	(Huang and Chen, 2018)
	Tomato	SSC, pH	PLS	SSC: r_p_=0.800, pH: r_p_=0.819	(Huang et al., 2018a)
	Tomato	Firmness, SSC, pH	PLS	Firmness: R=0.835, SSC: R=0.623, pH: R=0.769	(Huang et al., 2018b)
	Tomato	Firmness, puncture maximum force, slope	PLS	F: 0.859, PMF: 0.917, SL: 0.948	(Huang et al., 2018c)
	Tomato	Maturity stages	SVMDA	Total classification accuracy=98.3%	(Huang et al., 2020a)
	Tomato	Ripeness	PLS-DA	Classification accuracy=88.4%	(Zhu et al., 2015)
**Wood**	Softwood silver fir	Dry, wet	MC	Dry: µ_a_=0.0048mm^-1^, 0.0042mm^-1^, µ_s_’=1.8mm^-1^, 13 mm^-1^, Wet: µ_a_=0.0045mm^-1^, 0.0038mm^-1^, µ_s_’=0.6mm^-1^, 2.0mm^-1^	(Kienle et al., 2008)
	Douglas fir	Various densities, grain directions, thicknesses	PCR, PLS	3mm: R=0.953, 5mm: R=0.987	(Ma et al., 2018)
	Five softwood (SW) ten hardwood (HW)	Classification	PCA, QDA	QDA=94.0%	(Ma et al., 2019a)
	Hinoki cypress	Three-dimensional grain angle	GPR, LRA	GPR: R^2^ = 0.98, RMSE=2.2° LRA: R^2^>0.90, RMSE<3.8°	(Ma et al., 2019b)
	Wood	Tensile strain measurement	PCA, PLSR	R^2^ = 0.86, RMSE=279.86	(Ma et al., 2021c)
	Wood	Classification	PCA, SVM	Five-fold cross-validation=98.6%, Test set validation=91.2%	(Ma et al., 2021a)
**Animal** **Husbandry**	Cattle	Age	PLS, GA, RLT	ARMSEP=2.0 years, R^2^ = 0.63	(Palendeng et al., 2020)
	Rabbit	Early pregnancy diagnosis	PLS-DA, CARS, SPA, SPA, SVM, KNN, Naïve Bayes	Validation set Sensitivity=93.18%, Specificity=94.44%, Accuracy=93.88%, Prediction set Sensitivity=86.96%, Specificity=90.00%, Accuracy=90.69%	(Yuan et al., 2022)

JVSPO, Joint variable selection and preprocessing optimization method; MLD, Modified Lorentzian distribution; GD, ‘Golden Delicious’; RD, ‘Delicious’ (RD) apples; ANOVA, Analysis of variance; LSD, Least significant difference; LDA, Linear discriminant analysis; C-SVC, C-Support Vector Classification algorithm; GL, Gaussian-Lorentzian cross product; r_p_, Correlation coefficient of prediction; PCR, Principal component regression; PLS, Partial least squares regression analysis; GPR, Gaussian process regression; RMSE, Root mean square error; LRA, Linear regression analysis; PCA, Principal component analysis; QDA, Quadratic discriminant analysis; PLS, Partial least squares regression; GA, Genetic algorithm; RLT, Repeated learning-training method; ARMSEP, Average root mean square error of prediction; MC, Monte Carlo simulations; PLS-DA, Partial least squares-discriminant analysis; CARS, Competitive adaptive reweighted sampling; SPA, Successive projection algorithm; SVM, Support vector machine; KNN, K-Nearest Neighbor.

### 4.2 Applications of meat products field

In the detection of meat products, Xia et al. applied the SRS technique to the detection of meat products for the first time. They measured the SRS of beef samples with a single-fiber detection, obtained the absorption coefficient and scattering coefficient of beef through the diffuse reflectance equation, and established a correlation analysis between beef shear force and scattering coefficient, with a coefficient of determination (R^2^) of 0.59, which verified the feasibility of SRS in detecting beef tenderness (Xia et al., 2007; Xia et al., 2008). Zhang et al. studied the tenderness of pork using multi-channel SRS and predicted the tenderness of pork by decreasing the scattering coefficient, which was R^2^ = 0.8349 for fresh meat shear, through which the tenderness of pork can be directly predicted to realize fast and non-destructive detection (Zhang et al., 2010). Wen et al. investigated myoglobin content in pork and found that SRS in the short wave range was a feasible method for detecting myoglobin content with a significant correlation R^2^ = 0.955 (Wen et al., 2010). Wang et al. determined the moisture content of the complete pork using SRS and found that the steady-state SRS was capable of significantly forecasting the moisture content of the pork compared to the conventional Y-fiber, with an R^2^ of 0.8078 for their model. (Wang J. et al., 2017).

In summary, SRS is currently applied to detect tenderness, myoglobin and moisture content of meat products. Moreover, this technique can improve the accuracy of meat quality prediction to a great extent. **Table 2** summarizes in detail the results of research on meat product quality testing. While relatively few meat products can be tested by this method, SRS has excellent potential for future applications in meat quality testing.

### 4.3 Applications of fruit and vegetable field

SRS is widely applied in fruit inspection, mainly for apples, pears, peaches, kiwifruit, bananas, and citrus. In the detection process of apples, spatially resolved hyperspectral imaging was to measure apple OP and relate them to fruit firmness and SSC, showing that the *µ_a_* and *µ_s_’* data gave the best predictions for the fruit firmness and SSC, with correlation coefficients (*r*) of 0.82 and 0.80 for firmness, and 0.7 and 0.59 for SSC respectively. This provides a fresh approach to detecting the internal quality of fruits (Qin et al., 2007). Peng and Lu refined the hyperspectral scattering technique for fruit quantity testing by fitting spectral scattering curves at each wavelength with ten different forms of modified Lorentzian distribution functions and comparing the predictions of fruit firmness and SSC by ten modified Lorentzian distribution functions using multiple linear regression and cross-validation methods. The predicted correlation coefficients were 0.894 and 0.883, respectively, which verified the advantages of the technique in fruit quality testing (Peng and Lu, 2008). Lu et al. used the absorption scattering properties of apple tissue to predict bruising of the fruit. The measurement of enhanced scattering properties was found to be feasible for bruise detection in apples (Lu et al., 2010). Huang et al. detected the mealiness of apples, modeled the classification of apple mealiness classes by the partial least squares (PLS) method, and found that the accuracy of establishing a two-level classification was ≥93%. Thus, it validated the advantages of hyperspectral scattering technology in the nondestructive detection of the mealiness of apples (Huang and Lu, 2010). Cen et al. analyzed the physical and structural properties of apple pulp using a newly developed OPA (Cen et al., 2012b) and the correlation coefficients of firmness, *r*=0.870-0.948, and Young’s modulus, *r*=0.585-0.947, were obtained for Golden Delicious (GD) apples, which demonstrated that spatially resolved techniques can be used to predict internal fruit quality by combining OP (Cen et al., 2013). Mendoza et al. used short-wave NIR spectroscopy and scattering to classify apple quality with accuracies ranging from 87.3-97.6% for firmness and 77.1-92.3% for SSC, which validated the capability of organizing and grading apples by firmness and SSC (Mendoza et al., 2014). Nguyen Do Trong obtained the scattering and absorption coefficients of apple slices air-dried under various conditions pretreated by spatially resolved diffuse reflectance spectroscopy. Finally, it was found that SRS could detect the microstructure and quality relationship of air-dried apple slices without loss (Nguyen Do Trong et al., 2014b). The spatially resolved diffuse reflectance device (Nguyen Do Trong et al., 2013) was used to detect the OP of apples (Nguyen Do Trong et al., 2014a). The *μ_a_* spectrum was found to be superior to *μ_s_’* by comparison, and the coefficients of determination R^2^ for firmness and SSC were 0.71 and 0.81, respectively. The results showed that the detection of diffuse reflectance spectra of optical fibers cannot significantly improve the prediction performance of SSC. Still, it can be used to better predict the firmness and SSC of apples by separating the absorption coefficients and reducing the scattering coefficients. Zhu et al. utilized hyperspectral scattering to expected damage to apples with predictive correlation coefficients R_p_=0.848-0.919. The research revealed that hyperspectral scattering can be used to assess the bruise susceptibility of apples, which is beneficial for post-harvest inspection of fruits (Zhu et al., 2016). Mollazade et al. found a way to classify apple fruits using spatial resolving technique, which was verified by 76% and 82% accuracy for non-mealy and mealy apples, respectively (Mollazade and Arefi, 2017). Huang et al. used a multichannel HSI to classify apple varieties with 99.4% accuracy using the best spectral classification. They verified the potential of multichannel hyperspectral imaging systems for apple variety detection (Huang et al., 2020b). Ma and Xia et al. used a multi-fiber, spatially resolved measurement system that assessed the SSC and firmness of apples with an optimal R^2^ of 0.97 and 0.96, respectively, validating the technique’s ability to detect apple quality in a low-cost and portable method accurately (Ma et al., 2021b). Ye et al. obtained spatially resolved interaction spectra at eight different source-detector distances (SDDs) on the fruit surface and verified that the optimal SD could be selected to detect the extent of red color in the flesh at a specific depth by a model developed for anthocyanin content estimation (Ye et al., 2021).

In the inspection of peaches, Cen et al. (2011; 2012a) measured the absorption and reduced scattering coefficients based on the SR method of HSI to assess peach ripeness and quality, with *r* of 0.749 and 0.504 for firmness and SSC, respectively. The results suggested that spatially resolved techniques had good potential for application. Research by Sun et al. measured the OP of peaches during quality damage, determined the relationship between optical parameters and specific structural and biochemical factors, and found a good correlation at 675 nm (Sun Y. et al., 2020). This study facilitated the early detection of peach diseases. Sun et al. also measured the OP of peaches at different ripeness levels using the SR technique (Sun Y. et al., 2021), with classification accuracies of 85% and 76.25%, respectively, and these results found that this optical property was effective in detecting damage in peaches. Huang et al. evaluated the firmness and SSC of peaches using SRS (Huang et al., 2022) and improved the prediction of peach quality by incorporating spectral disparity techniques, with the best *r* of peach firmness and SSC being 0.853 and 0.839, respectively. Joseph et al. used the SRS technique to study the relationship between peach porosity and light scattering characteristics, and the results showed that the reduced scattering coefficients at 760 nm and 835 nm were linearly correlated with the spatially averaged porosity by R^2^ of 0.66 and 0.57, respectively, which verified that the method could realize non-destructive pear porosity assessment (Joseph et al., 2023).

In addition, Ma et al. verified the feasibility of SRS for the detection of kiwifruit quality with coefficients of determination R^2^ of 0.81 and 0.59 for SSC and pH, respectively (Ma et al., 2022). The OP of the pear was analyzed by Hu et al. (2017). They measured *μ_a_* between 0.1-0.61 cm^-1^, while *μ_s_’* decreased with wavelength between 12.5-9.5 cm^-1^. In this study, it was demonstrated that the OP of pears is associated with the wavelength and that establishing standardized slices of the samples helps to enhance the precision of the measurement of the OP. Adebayo et al. combined OP with chlorophyll, modulus of elasticity, SSC, and banana ripeness to develop predictive models. The correlation coefficients of chlorophyll, elastic modulus, and SSC were 0.9768-0.9807, 0.9553-0.9759, and 0.9640-0.9801, respectively, and the classification accuracy of banana ripeness reached 97.53%. This indicates that bananas with different ripeness levels can be predicted and categorized by OP, which provides a good and effective method for nondestructive testing of banana quality (Adebayo et al., 2016). Lorente et al. predicted early decay in citrus fruits with a classification accuracy of 96.1%, validating that this technique has great potential for grading citrus fruits (Lorente et al., 2013).

In the detection of vegetables, Lu et al. used the spatially resolved technique of hyperspectral imaging to test for defective pickling cucumbers (Lu et al., 2011). They found that effective defect detection could be achieved by enhanced scattering characteristic measurements through analysis of the OP of cucumbers. Sun et al. developed the SR transmission spectroscopy system for detecting internal rot onions, and the presence of high area under curve (AUC) values (0.96 ± 0.02) and Kappa values (0.77 ± 0.05) at the stem end of the onion validated the advantages of the system in detecting onion decay (Sun J. et al., 2020). Huang et al. designed a multichannel SRS detection device and used it to detect firmness, SSC, pH with correlation coefficients of 0.835, 0.623, and 0.769, respectively (Huang et al., 2018a; Huang et al., 2018b), The classification accuracy in tomato maturity assessment was able to reach 98.3% (Huang et al., 2020a; Huang et al., 2020b), which verified that OP based on SRS can reasonably predict the quality of tomato.

**Table 2** shows the details of the studies on the detection of product quality of fruits and vegetables. It shows that SRS has been widely used in the field of fruits and vegetables, mainly for the detection of quality characteristics such as firmness, pH, SSC, maturity, mealiness and bruise, as well as the biochemical properties of the internal tissues. In the future, the application of SRS in fruit and vegetable detection will be more mature, the types of detection will be more abundant, and the accuracy will be higher.

### 4.4 Applications of forestry field

In the field of forestry industry, SRS is mainly used in the detection of wood in recent years. Kienle et al. used a spatially and time resolved approach to study the mechanism of light propagation in dry and moist softwoods and put forward a theoretical model for the description of light propagation in wood, which is supported by the microstructure of softwood (Kienle et al., 2008). Ma et al. used spatially resolved hyperspectral detection to examine the OP, grain direction, and thickness of Douglas-fir at different densities. Correlation coefficients for 3 mm and 5 mm samples were 0.953 and 0.987, respectively (Ma et al., 2018). Meanwhile, the device classified softwoods and hardwoods with an accuracy of 94.1%, which shows that SRS is highly predictive in wood inspection (Ma et al., 2019a). In addition, the SRS device was optimized to achieve 91.2% accuracy in the test set of 15 wood classifications (Ma et al., 2021a). Moreover, the R^2^ of the tensile strain of the wood was measured to be 0.86 using the optimized equipment, which verified the suitability of SRS for the detection of wood (Ma et al., 2021c).

**Table 2** summarizes in detail the current status of product quality testing in forestry. It can be found that SRS detection technology is emerging in the application of forestry quality testing, and with the progress of technology, this method will be more widely used in forestry-related testing.

### 4.5 Applications of animal husbandry field

In the field of animal husbandry, Palendeng et al. used a spatially resolved method to detect the age of cattle (Palendeng et al., 2020). The feasibility of the SR technique for estimating the age of cattle was validated by using the SR diffuse reflectance spectrometer based on a fiber optic probe to collect skin samples from the neck of the cattle and assessing the age of the cattle by the developed PLS model with the lowest average root mean square error of prediction (ARMSEP) of 2.0 years and R^2^ = 0.63. Yuan et al. used SRS to diagnose the possibility of pregnancy in female rabbits by collecting spectral information at different distances with a movable distance-type detection fiber (Yuan et al., 2022). The results showed that the SRS detection method can distinguish whether a female rabbit is pregnant or not, and the accuracy of the validation set can reach 90.69%.

From **Table 2**, it can be found that the application of SRS technology in animal husbandry-related fields is still rare, and it is currently only applied to a few animal husbandry tests, mainly for age and pregnancy detection of animals. However, this technique shows a strong predictive ability in livestock detection. Therefore, the method is expected to be widely applied to the detection of other characteristics of animal husbandry and more livestock animals in future development.

Generally, SRS technology has been widely used in the field of agriculture. In the field of dairy this technique is mainly applied to the detection of protein, fat and other nutrients in milk with better predicted results. But at present the technique is less used for the detection of other types of dairy products such as goat milk, camel milk, etc., and some dairy products such as milk powder, cream, cheese, etc. Hence there is a good prospect for development in this field. In the detection of meat products, researchers mainly focus on the detection of fresh beef and pork, and the qualities detected are meat tenderness, myoglobin and moisture content. Nevertheless, the quality of some meat products such as jerky, dried meat, bacon, sausage, etc. was less tested. In the future, other types of meat can also be detected, such as lamb, fish, shrimp, etc., through the detection of its nutrient content to predict the quality of meat products, which is conducive to providing human beings with more healthy and nutritious food. SRS has been most used and developed for fruits and vegetables. Currently, the fruits and vegetables inspected include apples, pears, kiwifruits, bananas, citrus, cucumbers, onions, and tomatoes. The main detection of their SSC, hardness, pH, damage, ripeness, chlorophyll content, etc. Thus, SRS is expected to achieve more efficient and accurate quality detection in this field. In the field of forestry, researchers mainly apply SRS to the classification, moisture, texture, and thickness detection of wood. In future development, it is expected to realize the detection of hardness, oiliness, density and damage of wood, which has a lot of space for development. In the field of animal husbandry, the current research is mainly focused on the age of cows and the pregnancy of rabbits, but in the future, it is expected to detect more animals and their health level. Overall, SRS has been more widely used in the field of agriculture at present but still has a lot of advantages for development. Since this technique can not only directly analyze the correlation through spatial spectral information, but also extract specific optical properties to further explore the relationship between the quality of agricultural products and OP. Therefore, it is expected that this technology will have much more effective application potential in future quality detection in the field of agriculture.

## 5 Challenges and future trends

SRS has been widely used in agriculture so far due to its stable performance, low cost, ease of use, and continuous algorithmic improvement. Importantly, this is mainly because that detection method can well reflect the characteristics of agricultural products. Although five different types of SRS, including single fiber, fiber array type, CCD line scan type, hyperspectral line-scan, and multi-channel hyperspectral imaging detection system, are relatively widely used in agricultural products for quality inspection currently, this technology still faces many challenges and difficulties.

The challenges are mainly in the SRS devices and calculation methods. In terms of devices, for example, there is no specific standard for the selection of light sources, and the selection of high-power light sources can easily damage the external and internal tissue structure of organisms. In contrast, the selection of low-power light sources has limited detection distance and cannot collect satisfactory distant spectral information. To meet the requirement of detection, the light source should satisfy the appropriate intensity meanwhile its diameter is often small enough, especially for small samples such as corn kernels, wheat seeds, cherries, grapes, and other agricultural products, so that it can be equivalent to a point light source and reduce the error of solving the OP of the calculation. The practical arrangement and selection of optical fiber is also a problematic issue in the device. For single fiber and fiber array detection devices, the selection of optical fiber is significant, which not only requires the fiber to be as small as possible but also the arrangement of the detection distance as accurately as possible. Besides, for irregular detection objects, the detection fibers often cannot fit closely due to the curvature of the sample surface. Although Huang et al. designed a multi-channel detection device, the approach is only suitable for larger objects with micro-curvature. Some irregular-shaped and curvature-changed objects or smaller objects still cannot be detected satisfactorily. Therefore, how to design a detection fiber that can meet irregular objects is still an essential and inevitable challenge.

In addition, the stability and precision of the mechanical device enable more accurate acquisition of spatially resolved spectral information. Therefore, the design of the mechanics of the SRS is of great significance. During the spectra acquisition process, the detection device needs to hold the fiber and the sample firmly in place. Manual detection often lacks accuracy, which is prone to jitter, and has many instabilities. These can undoubtedly have a negative impact on the spectral quality. As a result, more stable mechanical devices are needed to replace manual fixation to improve the stability of the detection system. However, the mechanical device has different requirements for the detection fiber, light source, and sample. As for optical fiber detection, it is not only required that the fiber closely fits the sample but also that the fiber is moved in a more precise position. However, because of the irregularity of the measured object and the curvature of the surface, it is easy for the optical fibers to move without close contact, and can also damage the surface of the agricultural product if it is moved too aggressively. In the case of the light source, it is necessary that the light source is also close-fitting the measured object surface to avoid too much diffuse light on the spectral information. As for the sample, the mechanical device should be fixed steadily so that the collected sample cannot move easily, and it should not be fixed too tightly to avoid damage or deformation to the sample. So, there are many difficulties in the design of the mechanical device.

Moreover, the detection accuracy has a great impact on the subsequent analysis of the spectra. Trying to minimize the impact of some controllable factors on the accuracy is beneficial to improving the spectral detection quality. The detection accuracy is affected by various factors, which are reflected in all aspects of the detection device. For example, the resolution of the detection instrument, the stability of the light source, the loss of the detection fiber, and the stability of the mechanical device might have a negative impact on the accuracy. Therefore, the designed SRS equipment requires calibration to guarantee the stability and accuracy of the equipment. Last but not least, the design cost of SRS is also a problem because the manufacturing and maintenance costs of SRS detection devices are very high and usually require the use of expensive optical and mechanical components. How to improve the detection accuracy of SRS while reducing the cost is also a demanding challenge to be solved.

At the present stage, direct analysis and OP are mainly used to deal with spatially resolved spectra in terms of computational methods. The direct analysis method is simple and efficient, but this method is less used. Therefore, it is a promising trend and a good development direction to study the simple and efficient direct analysis method. In addition, the OP method is complex, but the prediction accuracy is relatively high. The current OP methods are based on the diffuse equation theory to separate out the optical characteristics. In the process of extracting the OP, because of the complexity of the diffuse equation, the solved values are often not accurate enough and the computation is huge. The future development of simpler and more accurate optical equations to extract OP based on the current research is also an emerging research prevailing trend.

## 6 Conclusion

Agricultural products, including dairy, meat, fruit and vegetable, forestry products, and animal husbandry products, are of great importance to people’s daily lives, depending on their external and internal quality. Compared with traditional detection methods, SRS not only provides more spatial information but also separates out optical properties, so it can be widely used in the field of agriculture. SRS detection systems, including single fiber, fiber array type, CCD line scan type, hyperspectral line-scan, and multi-channel hyperspectral imaging detection system, have been increasingly used for inspecting quality in replacement of manual grading as they can provide a simple structure, easy to operate, low cost and non-destructive assessment. With the continuous development of this technology, many successful applications have proved that SRS detection systems are powerful and scientific tools for stable and accurate quality inspection of agricultural products. This paper reviews the principles, development, and applications of five various SRS detection systems for agricultural product quality inspections. Despite the problems and challenges of this technique, it promises to achieve online detection with a more simple, portable, and easy to operate configuration for further widespread application in quality inspection of agricultural fields.

## Author contributions

YX: Writing – original draft, Writing – review & editing, Conceptualization, Methodology, Funding acquisition, Supervision. WXL: Writing – original draft, Writing – review & editing, Conceptualization, Investigation, Data curation, Methodology. JM: Data curation, Software, Writing – original draft. JH: Formal Analysis, Writing – original draft. WBL: Writing – review & editing, Resources. JK: Writing – review & editing, Funding acquisition, Project administration. BL: Writing – review & editing, Resources. HZ: Writing – review & editing, Resources, Supervision. WT: Writing – review & editing, Resources, Supervision.

## References

[B1] Adebayo S. E. Hashim N. Abdan K. Hanafi M. Mollazade K. (2016). Prediction of quality attributes and ripeness classification of bananas using optical properties. Scientia Hortic. 212, 171–182. doi: 10.1016/j.scienta.2016.09.045

[B2] Audu J. Aremu A. K. (2021). Development, evaluation, and optimization of an automated device for quality detection and separation of cowpea seeds. Artif. Intell. Agric. 5, 240–251. doi: 10.1016/j.aiia.2021.10.003

[B3] Bao C. Zhang H. Li Q. Zhou S. Zhang H. Deng K.. (2021). Spatially-resolved electronic structure of stripe domains in IrTe2 through electronic structure microscopy. Commun. Phys. 4, 229. doi: 10.1038/s42005-021-00733-x

[B4] Bogomolov A. Belikova V. Galyanin V. Melenteva A. Meyer H. (2017). Reference-free spectroscopic determination of fat and protein in milk in the visible and near infrared region below 1000nm using spatially resolved diffuse reflectance fiber probe. Talanta 167, 563–572. doi: 10.1016/j.talanta.2017.02.047 28340762

[B5] Bridger K. G. Roccabruna J. R. Baran T. M. (2021). Optical property recovery with spatially-resolved diffuse reflectance at short source-detector separations using a compact fiber-optic probe. Biomed. Opt. Express 12, 7388–7404. doi: 10.1364/BOE.443332 35003841 PMC8713658

[B6] Cen H. Lu R. (2009). Quantification of the optical properties of two-layer turbid materials using a hyperspectral imaging-based spatially-resolved technique. Appl. Opt. 48, 5612–5623. doi: 10.1364/ao.48.005612 19823246

[B7] Cen H. Lu R. (2010). Optimization of the hyperspectral imaging-based spatially-resolved system for measuring the optical properties of biological materials. Opt. Express 18, 17412–17432. doi: 10.1364/OE.18.017412 20721128

[B8] Cen H. Lu R. A. Mendoza F. P. Ariana D. (2012a). Assessing multiple quality attributes of peaches using optical absorption and scattering properties. Trans. ASABE 55, 647–657. doi: 10.13031/2013.41366

[B9] Cen H. Lu R. Dolan K. (2010). Optimization of inverse algorithm for estimating the optical properties of biological materials using spatially-resolved diffuse reflectance. Inverse Problems Sci. Eng. 18, 853–872. doi: 10.1080/17415977.2010.492516

[B10] Cen H. Lu R. Mendoza F. A. (2012b). Analysis of absorption and scattering spectra for assessing the internal quality of apple fruit. Acta Hortic. 945, 181–188. doi: 10.17660/ActaHortic.2012.945.24

[B11] Cen H. Lu R. Mendoza F. A. Ariana D. P. (2011). Peach maturity/quality assessment using hyperspectral imaging-based spatially resolved technique, in Proceedings of SPIE 8027, Sensing for Agriculture and Food Quality and Safety III, (Bellingham, USA: 80270L, Society of Photo-optical Instrumentation Engineers). doi: 10.1117/12.883573

[B12] Cen H. Lu R. Mendoza F. Beaudry R. M. (2013). Relationship of the optical absorption and scattering properties with mechanical and structural properties of apple tissue. Postharvest Biol. Technol. 85, 30–38. doi: 10.1016/j.postharvbio.2013.04.014

[B13] Chen Y. Xu Z. Tang W. Hu M. Tang D. Zhai G.. (2021). Identification of various food residuals on denim based on hyperspectral imaging system and combination optimal strategy. Artif. Intell. Agric. 5, 125–132. doi: 10.1016/j.aiia.2021.06.001

[B14] Cheong W. F. Prahl S. A. Welch A.J.J.I.J.O.Q.E. (1990). A review of the optical properties of biological tissues. IEEE J. Quantum Electron. 26, 2166–2185. doi: 10.1109/3.64354

[B15] Chong K. C. Pramanik M. (2023). Physics-guided neural network for tissue optical properties estimation. Biomed. Opt. Express 14, 2576–2590. doi: 10.1364/BOE.487179 37342718 PMC10278626

[B16] Colas V. Amouroux M. Perrin-Mozet C. Daul C. Blondel W. (2023). Photometric and Monte-Carlo modeling unified approach for the calculation of spatially-resolved correction coefficients linking simulated and experimental diffuse reflectance spectra. Opt. Express 31, 25954–25969. doi: 10.1364/OE.491921 37710468

[B17] Comerford J. M. Negus J. Barrows R. S. Wylezalek D. Greene J. E. Müller-Sánchez F.. (2022). Toward a more complete optical census of active galactic nuclei via spatially resolved spectroscopy. Astrophys J. 927, 23. doi: 10.3847/1538-4357/ac496a

[B18] Cubeddu R. D’andrea C. Pifferi A. Taroni P. Torricelli A. Valentini G.. (2001). Nondestructive quantification of chemical and physical properties of fruits by time-resolved reflectance spectroscopy in the wavelength range of 650–1000 nm. Appl. Opt. 40, 538–543. doi: 10.1364/ao.40.000538 18357029

[B19] Dam J. S. Pedersen C. B. Dalgaard T. Fabricius P. E. Aruna P. Andersson-Engels S. (2001). Fiber-optic probe for noninvasive real-time determination of tissue optical properties at multiple wavelengths. Appl. Opt. 40, 1155–1164. doi: 10.1364/ao.40.001155 18357101

[B20] De Man A. Uyttersprot J. S. Chavez P. F. Vandenbroucke F. Bovart F. De Beer T. (2023). The application of Near-Infrared Spatially Resolved Spectroscopy in scope of achieving continuous real-time quality monitoring and control of tablets with challenging dimensions. Int. J. Pharm. 641, 123064. doi: 10.1016/j.ijpharm.2023.123064 37211236

[B21] Dhanya V. G. Subeesh A. Kushwaha N. L. Vishwakarma D. K. Nagesh Kumar T. Ritika G.. (2022). Deep learning based computer vision approaches for smart agricultural applications. Artif. Intell. Agric. 6, 211–229. doi: 10.1016/j.aiia.2022.09.007

[B22] Doornbos R. M. Lang R. Aalders M. C. Cross F. W. Sterenborg H. J. (1999). The determination of in *vivo* human tissue optical properties and absolute chromophore concentrations using spatially resolved steady-state diffuse reflectance spectroscopy. Phys. Med. Biol. 44, 967–981. doi: 10.1088/0031-9155/44/4/012 10232809

[B23] Farrell T. J. Patterson M. S. Wilson B. (1992). A diffusion theory model of spatially resolvedsteady-state diffuse reflectance for the non-invasive determination of tissue optical properties in *vivo* . Med. Phys. 19, 879–888. doi: 10.1118/1.596777 1518476

[B24] Guo Z. Zhang Y. Wang J. Liu Y. Jayan H. El-Seedi H. R.. (2023). Detection model transfer of apple soluble solids content based on NIR spectroscopy and deep learning. Comput. Electron. Agric. 212, 108127. doi: 10.1016/j.compag.2023.108127

[B25] Hank P. Blum C. Liemert A. Geiger S. Kienle A. (2023). Analytical solution for the single scattered radiance of two-layered turbid media in the spatial frequency domain. Part 2: Vector radiative transfer equation. Optics Commun. 535, 129354. doi: 10.1016/j.optcom.2023.129354

[B26] Haskell R. C. Svaasand L. O. Tsay T. T. Feng T. C. Mcadams M. S. Tromberg B. J. (1994). Boundary conditions for the diffusion equation in radiative transfer. Opt Soc. Am. A Opt Image Sci. Vis. 11, 2727–2741. doi: 10.1364/josaa.11.002727 7931757

[B27] Hu D. Fu X. Ying Y. (2017). Characterizing pear tissue with optical absorption and scattering properties using spatially-resolved diffuse reflectance. J. Food Meas. Charact. 11, 930–936. doi: 10.1007/s11694-017-9465-x

[B28] Hu D. Lu R. Ying Y. (2018). A two-step parameter optimization algorithm for improving estimation of optical properties using spatial frequency domain imaging. J. Quant. Spectrosc. Radiat. Transf. 207, 32–40. doi: 10.1016/j.jqsrt.2017.12.022

[B29] Hu D. Lu R. Ying Y. (2020a). Spatial-frequency domain imaging coupled with frequency optimization for estimating optical properties of two-layered food and agricultural products. J. Food Eng. 277, 109909. doi: 10.1016/j.jfoodeng.2020.109909

[B30] Hu D. Sun T. Yao L. Yang Z. Wang A. Ying Y. (2020b). Monte Carlo: A flexible and accurate technique for modeling light transport in food and agricultural products. Trends Food Sci. Technol. 102, 280–290. doi: 10.1016/j.tifs.2020.05.006

[B31] Huang R. L. Q. C. Chen K.-J. (2018). Tomato maturity classification based on spatially resolved spectra. Spectrosc. Spectral Anal. 38, 2183–2188. doi: 10.3964/j.issn.1000-0593(2018)07-2183-06

[B32] Huang M. Lu R. (2010). Apple mealiness detection using hyperspectral scattering technique. Postharvest Biol. Technol. 58, 168–175. doi: 10.1016/j.postharvbio.2010.08.002

[B33] Huang Y. Lu R. Chen K. (2017). Development of a multichannel hyperspectral imaging probe for property and quality assessment of horticultural products. Postharvest Biol. Technol. 133, 88–97. doi: 10.1016/j.postharvbio.2017.07.009

[B34] Huang Y. Lu R. Chen K. (2018a). Assessment of tomato soluble solids content and pH by spatially-resolved and conventional Vis/NIR spectroscopy. J. Food Eng. 236, 19–28. doi: 10.1016/j.jfoodeng.2018.05.008

[B35] Huang Y. Lu R. Hu D. Chen K. (2018b). Quality assessment of tomato fruit by optical absorption and scattering properties. Postharvest Biol. Technol. 143, 78–85. doi: 10.1016/j.postharvbio.2018.04.016

[B36] Huang Y. Lu R. Xu Y. Chen K. (2018c). Prediction of tomato firmness using spatially-resolved spectroscopy. Postharvest Biol. Technol. 140, 18–26. doi: 10.1016/j.postharvbio.2018.02.008

[B37] Huang Y. Si W. Chen K. Sun Y. (2020a). Assessment of tomato maturity in different layers by spatially resolved spectroscopy. Sens. (Basel) 20, 7229. doi: 10.3390/s20247229 PMC7766491 33348611

[B38] Huang Y. Xiong J. Jiang X. Chen K. Hu D. (2022). Assessment of firmness and soluble solids content of peaches by spatially resolved spectroscopy with a spectral difference technique. Comput. Electron. Agric. 200, 107212. doi: 10.1016/j.compag.2022.107212

[B39] Huang Y. Yang Y. Sun Y. Zhou H. Chen K. (2020b). Identification of apple varieties using a multichannel hyperspectral imaging system. Sens. (Basel) 20, 5120. doi: 10.3390/s20185120 PMC7571201 32911790

[B40] Igne B. Talwar S. Feng H. Drennen J. K. Anderson C. A. (2015). Near-infrared spatially resolved spectroscopy for tablet quality determination. J. Pharm. Sci. 104, 4074–4081. doi: 10.1002/jps.24618 26317576

[B41] Joseph M. Van Cauteren H. Postelmans A. Nugraha B. Verreydt C. Verboven P.. (2023). Porosity quantification in pear fruit with X-ray CT and spatially resolved spectroscopy. Postharvest Biol. Technol. 204, 112455. doi: 10.1016/j.postharvbio.2023.112455

[B42] Kalinin A. V. Krasheninnikov V. N. Krivtsun V. M. (2013). Short-wave near infrared spectrometry of back scattering and transmission of light by milk for multi-component analysis. J. Near Infrared Spectrosc. 21, 35–41. doi: 10.1255/jnirs.1034

[B43] Katsumata K. (2022). A theory of reflection and refraction of light by a metal. Optical Rev. 29, 159–171. doi: 10.1007/s10043-022-00732-5

[B44] Kienle A. D’andrea C. Foschum F. Taroni P. Pifferi A. (2008). Light propagation in dry and wet softwood. Opt. Express 16, 9895–9906. doi: 10.1364/oe.16.009895 18575559

[B45] Kienle A. Lilge L. Patterson M. S. Hibst R. Steiner R. Wilson B. C. (1996). Spatially resolved absolute diffuse reflectance measurements for noninvasive determination of the optical scattering and absorption coefficients of biological tissue. Appl. Opt. 35, 2304–2314. doi: 10.1364/ao.35.002304 21085367

[B46] Kienle A. Patterson M. S. (1997). Improved solutions of the steady-state and the time-resolved diffusion equations for reflectance from a semi-infinite turbid medium. Opt Soc. Am. A Opt. Image Sci. Vis. 14, 246–254. doi: 10.1364/josaa.14.000246 8988618

[B47] Kienle A. Patterson M. S. Dögnitz N. Bays R. Wagniνres G. Van Den Bergh H. (1998). Noninvasive determination of the optical properties of two-layered turbid media. Appl. Opt. 37, 779–791. doi: 10.1364/ao.37.000779 18268653

[B48] Kubelka P. (1948). New contributions to the optics of intensely light-scattering materials. Part I. J. Optical Soc. America 38, 448–457. doi: 10.1364/JOSA.38.000448 18916891

[B49] Lan Q. Mcclarren R. G. Vishwanath K. (2023). Neural network-based inverse model for diffuse reflectance spectroscopy. Biomed. Opt. Express 14, 4725–4738. doi: 10.1364/BOE.490164 37791254 PMC10545200

[B50] Lee J. H. Kim S. Kim Y. T. (2004). Finite element method for diffusive light propagations in index-mismatched media. Opt. Express 12 8, 1727–1740. doi: 10.1364/opex.12.001727 19474999

[B51] Li M. Gu J. Zhang D. Gao Q. Li B. (2021). Equivalence ratio measurements in CH4/air gases based on the spatial distribution of the emission intensity of femtosecond laser-induced filament. Processes 9, 2022. doi: 10.3390/pr9112022

[B52] Liu G. Asif M. Mohri K. Schulz C. Dreier T. Endres T.. (2022). *In situ* measurement of gas-borne silicon nanoparticle volume fraction and temperature by spatially and spectrally line-resolved attenuation and emission imaging. Powder Technol. 396, 535–541. doi: 10.1016/j.powtec.2021.11.017

[B53] Lorente D. Zude M. Regen C. Palou L. Gómez-Sanchis J. Blasco J. (2013). Early decay detection in citrus fruit using laser-light backscattering imaging. Postharvest Biol. Technol. 86, 424–430. doi: 10.1016/j.postharvbio.2013.07.021

[B54] Lu R. Ariana D. P. Cen H. (2011). Optical absorption and scattering properties of normal and defective pickling cucumbers for 700–1000 nm. Sens. Instrument. Food Qual. Saf. 5, 51–56. doi: 10.1007/s11694-011-9108-6

[B55] Lu R. Cen H. Huang M. Ariana D.P.J.T.O.T.A. (2010). Spectral absorption and scattering properties of normal and bruised apple tissue. Trans. ASABE 53, 263–269. doi: 10.13031/2013.29491

[B56] Lu R. Van Beers R. Saeys W. Li C. Cen H. (2020). Measurement of optical properties of fruits and vegetables: A review. Postharvest Biol. Technol. 159, 111003. doi: 10.1016/j.postharvbio.2019.111003

[B57] Ma T. Inagaki T. Ban M. Tsuchikawa S. (2019a). Rapid identification of wood species by near-infrared spatially resolved spectroscopy (NIR-SRS) based on hyperspectral imaging (HSI). Holzforschung 73, 323–330. doi: 10.1515/hf-2018-0128

[B58] Ma T. Inagaki T. Tsuchikawa S. (2019b). Three-dimensional grain angle measurement of softwood (Hinoki cypress) using near infrared spatially and spectrally resolved imaging (NIR-SSRI). Holzforschung 73, 817–826. doi: 10.1515/hf-2018-0273

[B59] Ma T. Inagaki T. Tsuchikawa S. (2021a). Demonstration of the applicability of visible and near-infrared spatially resolved spectroscopy for rapid and nondestructive wood classification. Holzforschung 75, 419–427. doi: 10.1515/hf-2020-0074

[B60] Ma T. Inagaki T. Yoshida M. Ichino M. Tsuchikawa S. (2021c). Measuring the tensile strain of wood by visible and near-infrared spatially resolved spectroscopy. Cellulose 28, 10787–10801. doi: 10.1007/s10570-021-04239-1

[B61] Ma T. Schajer G. Inagaki T. Pirouz Z. Tsuchikawa S. (2018). Optical characteristics of Douglas fir at various densities, grain directions and thicknesses investigated by near-infrared spatially resolved spectroscopy (NIR-SRS). Holzforschung 72, 789–796. doi: 10.1515/hf-2017-0213

[B62] Ma T. Xia Y. Inagaki T. Tsuchikawa S. (2021b). Rapid and nondestructive evaluation of soluble solids content (SSC) and firmness in apple using Vis–NIR spatially resolved spectroscopy. Postharvest Biol. Technol. 173, 111417. doi: 10.1016/j.postharvbio.2020.111417

[B63] Ma T. Zhao J. Inagaki T. Su Y. Tsuchikawa S. (2022). Rapid and nondestructive prediction of firmness, soluble solids content, and pH in kiwifruit using Vis–NIR spatially resolved spectroscopy. Postharvest Biol. Technol. 186, 111841. doi: 10.1016/j.postharvbio.2022.111841

[B64] Martelli F. Tommasi F. Fini L. Cortese L. Sassaroli A. Cavalieri S. (2021). Invariance properties of exact solutions of the radiative transfer equation. J. Quant. Spectrosc. Radiat. Transf. 276, 107887. doi: 10.1016/j.jqsrt.2021.107887

[B65] Mei M. Li J. (2023). An overview on optical non-destructive detection of bruises in fruit: Technology, method, application, challenge and trend. Comput. Electron. Agric. 213, 108195. doi: 10.1016/j.compag.2023.108195

[B66] Mendoza F. Lu R. Ariana D. Cen H. Bailey B. (2011). Integrated spectral and image analysis of hyperspectral scattering data for prediction of apple fruit firmness and soluble solids content. Postharvest Biol. Technol. 62, 149–160. doi: 10.1016/j.postharvbio.2011.05.009

[B67] Mendoza F. Lu R. Cen H. (2014). Grading of apples based on firmness and soluble solids content using Vis/SWNIR spectroscopy and spectral scattering techniques. J. Food Eng. 125, 59–68. doi: 10.1016/j.jfoodeng.2013.10.022

[B68] Mishra P. Nordon A. Mohd Asaari M. S. Lian G. Redfern S. (2019). Fusing spectral and textural information in near-infrared hyperspectral imaging to improve green tea classification modelling. J. Food Eng. 249, 40–47. doi: 10.1016/j.jfoodeng.2019.01.009

[B69] Mohd Ali M. Hashim N. Bejo S. K. Jahari M. Shahabudin N. A. (2023). Innovative non-destructive technologies for quality monitoring of pineapples: Recent advances and applications. Trends Food Sci. Technol. 133, 176–188. doi: 10.1016/j.tifs.2023.02.005

[B70] Mollazade K. Arefi A. (2017). Optical analysis using monochromatic imaging-based spatially-resolved technique capable of detecting mealiness in apple fruit. Scientia Hortic. 225, 589–598. doi: 10.1016/j.scienta.2017.08.005

[B71] Morimoto K. Iguchi A. Tsuji Y. (2020). Propagation operator based boundary condition for finite element analysis. IEEE Photonics J. 12, 1–13. doi: 10.1109/JPHOT.2020.3015498

[B72] Nguyen Do Trong N. Erkinbaev C. Nicolaï B. M. Saeys W. Tsuta M. De Baerdemaeker J. (2013). Spatially resolved spectroscopy for nondestructive quality measurements of Braeburn apples cultivated in sub-fertilization condition, in Proceedings of SPIE 8881, Sensing Technologies for Biomaterial, Food, and Agriculture. (Bellingham, USA: 88810L, Society of Photo-optical Instrumentation Engineers). doi: 10.1117/12.2030407

[B73] Nguyen Do Trong N. Erkinbaev C. Tsuta M. De Baerdemaeker J. Nicolaï B. Saeys W. (2014a). Spatially resolved diffuse reflectance in the visible and near-infrared wavelength range for non-destructive quality assessment of ‘Braeburn’ apples. Postharvest Biol. Technol. 91, 39–48. doi: 10.1016/j.postharvbio.2013.12.004

[B74] Nguyen Do Trong N. Rizzolo A. Herremans E. Vanoli M. Cortellino G. Erkinbaev C.. (2014b). Optical properties–microstructure–texture relationships of dried apple slices: Spatially resolved diffuse reflectance spectroscopy as a novel technique for analysis and process control. Innovative Food Sci. Emerg. Technol. 21, 160–168. doi: 10.1016/j.ifset.2013.09.014

[B75] Nguyen Do Trong N. Watté R. Aernouts B. Herremans E. Verhoelst E. Tsuta M.. (2011). Spatially Resolved Spectroscopy for Non-destructive Quality Inspection of Foods (Louisville, Kentucky: ASABE), 1111381. doi: 10.13031/2013.37805

[B76] Nichols M. G. Hull E. L. Foster T. H. (1997). Design and testing of a white-light, steady-state diffuse reflectance spectrometer for determination of optical properties of highly scattering systems. Appl. Opt. 36, 93–104. doi: 10.1364/ao.36.000093 18250650

[B77] Niwayama M. Unno N. (2021). Tissue oximeter with selectable measurement depth using spatially resolved near-infrared spectroscopy. Sensors 21, 5573. doi: 10.3390/s21165573 34451015 PMC8402253

[B78] Palendeng M. E. Alvarenga T. I. R. C. Fowler S. Hopkins D. L. Mcgilchrist P. Thennadil S. N. (2020). Estimation of chronological age of cattle using spatially resolved diffuse reflectance measurements of hide. IEEE Sens. J. 20, 8673–8682. doi: 10.1109/jsen.2020.2983455

[B79] Peng Y. Lu R. (2008). Analysis of spatially resolved hyperspectral scattering images for assessing apple fruit firmness and soluble solids content. Postharvest Biol. Technol. 48, 52–62. doi: 10.1016/j.postharvbio.2007.09.019

[B80] Qin J. Lu R. (2007). Measurement of the absorption and scattering properties of turbid liquid foods using hyperspectral imaging. Appl. Spectrosc. 61, 388–396. doi: 10.1366/000370207780466190 17456257

[B81] Qin J. Lu R. Peng Y. (2007). Internal quality evaluation of apples using spectral absorption and scattering properties. SPIE Optics East 6761, 67610M. doi: 10.1117/12.751937

[B82] Qin J. Lu R. Peng Y. (2009). Prediction of apple internal quality using spectral absorption and scattering properties. Am. Soc. Agric. Biol. Engineers 52, 499–507. doi: 10.13031/2013.26807

[B83] Rejeb A. Rejeb K. Zailani S. Keogh J. G. Appolloni A. (2022). Examining the interplay between artificial intelligence and the agri-food industry. Artif. Intell. Agric. 6, 111–128. doi: 10.1016/j.aiia.2022.08.002

[B84] Reynolds L. Johnson C. Ishimaru A. (1976). Diffuse reflectance from a finite blood medium: applications to the modeling of fiber optic catheters. Appl. Optics 15, 2059–2067. doi: 10.1364/AO.15.002059 20165338

[B85] Ryo M. (2022). Explainable artificial intelligence and interpretable machine learning for agricultural data analysis. Artif. Intell. Agric. 6, 257–265. doi: 10.1016/j.aiia.2022.11.003

[B86] Sassaroli A. Tommasi F. Cavalieri S. Fini L. Liemert A. Kienle A.. (2022). Two-step verification method for Monte Carlo codes in biomedical optics applications. J. Biomed. Optics 27, 83018. doi: 10.1117/1.JBO.27.8.083018 PMC9020254 35445592

[B87] Sun C. Aernouts B. Van Beers R. Saeys W. (2021). Simulation of light propagation in citrus fruit using monte carlo multi-layered (MCML) method. J. Food Eng. 291, 110225. doi: 10.1016/j.jfoodeng.2020.110225

[B88] Sun B. Gao C. Spurr R. (2022). Scalar thermal radiation using the adding-doubling method. Opt. Express 30, 30075–30097. doi: 10.1364/OE.462580 36242119

[B89] Sun Y. Huang Y. Pan L. Wang X. (2021). Evaluation of the changes in optical properties of peaches with different maturity levels during bruising. Foods 10, 388. doi: 10.3390/foods10020388 33578918 PMC7916705

[B90] Sun J. Künnemeyer R. Mcglone A. Tomer N. Sharrock K. (2020). A spatially resolved transmittance spectroscopy system for detecting internal rots in onions. Postharvest Biol. Technol. 163, 111141. doi: 10.1016/j.postharvbio.2020.111141

[B91] Sun Y. Lu R. Pan L. Wang X. Tu K. (2020). Assessment of the optical properties of peaches with fungal infection using spatially-resolved diffuse reflectance technique and their relationships with tissue structural and biochemical properties. Food Chem. 321, 126704. doi: 10.1016/j.foodchem.2020.126704 32234637

[B92] Sun Z. Xie L. Hu D. Ying Y. (2021). An artificial neural network model for accurate and efficient optical property mapping from spatial-frequency domain images. Comput. Electron. Agric. 188, 106340. doi: 10.1016/j.compag.2021.106340

[B93] Tarasov A. P. Persheyev S. Rogatkin D. A. (2021). Analysis of the applicability of the classical probabilistic parameters of the Monte Carlo algorithm for problems of light transport in turbid biological media with continuous absorption and discrete scattering. Quantum Electron. 51, 408–414. doi: 10.1070/QEL17535

[B94] Tian X. Liu X. He X. Zhang C. Li J. Huang W. (2023). Detection of early bruises on apples using hyperspectral reflectance imaging coupled with optimal wavelengths selection and improved watershed segmentation algorithm. J. Sci. Food Agric. 103, 6689–6705. doi: 10.1002/jsfa.12764 37267465

[B95] Tian X. Zhang C. Li J. Fan S. Yang Y. Huang W. (2021). Detection of early decay on citrus using LW-NIR hyperspectral reflectance imaging coupled with two-band ratio and improved watershed segmentation algorithm. Food Chem. 360, 130077. doi: 10.1016/j.foodchem.2021.130077 34022516

[B96] Vanolia M. Van Beers R. Sadarc N. (2020). Time- and spatially-resolved spectroscopy to determine the bulk optical properties of ‘Braeburn’ apples after ripening in shelf life. Postharvest Biol. Technol. 168, 111233. doi: 10.1016/j.postharvbio.2020.111233

[B97] Vasudevan V. Narayanan Unni S.J.I.J.F.N.M.I.B.E. (2021). Quantification of soft tissue parameters from spatially resolved diffuse reflectance finite element models. Int. J. Numer Method BioMed. Eng. 38, e3546. doi: 10.1002/cnm.3546 34719121

[B98] Wang X. (2020). P3 equation steady-state model of light transport in semi-infinite thick rectangular medium. Optik 204, 164138. doi: 10.1016/j.ijleo.2019.164138

[B99] Wang X. (2022). P3 approximation equation of light transport in a slab medium: steady-state and time domains. Wave. Random Complex, 1–19. doi: 10.1080/17455030.2021.2014598

[B100] Wang Y. Coney C. Mcatee C. Mccullough G. Goguet A. (2022). Development of a spatially resolved technique for the measurement of effective diffusions and its application to the modelling of washcoated catalytic monoliths. Appl. Catalysis A: Gen. 638, 118608. doi: 10.1016/j.apcata.2022.118608

[B101] Wang J. Fan L. Wang H. Zhao P. Li H. Wang Z.. (2017). Determination of the moisture content of fresh meat using visible and near-infrared spatially resolved reflectance spectroscopy. Biosyst. Eng. 162, 40–56. doi: 10.1016/j.biosystemseng.2017.07.004

[B102] Wang L. Jacques S. (1992). Monte Carlo modeling of light transport in multi-layered tissues in standard C. (Houston, USA: The University of Texas, M. D. Anderson Cancer Center), 1–173.

[B103] Wang L. Jacques S. L. Zheng L. J. C. M. Biomedicine P. I. (1995). MCML—Monte Carlo modeling of light transport in multi-layered tissues. Comput. Methods Programs Biomed. 47, 131–146. doi: 10.1016/0169-2607(95)01640-F 7587160

[B104] Wang A. Lu R. Xie L. (2016). Finite element modeling of light propagation in turbid media under illumination of a continuous-wave beam. Appl. Opt. 55, 95–103. doi: 10.1364/ao.55.000095 26835627

[B105] Wang A. Lu R. Xie L. (2017). Improved algorithm for estimating the optical properties of food products using spatially-resolved diffuse reflectance. J. Food Eng. 212, 1–11. doi: 10.1016/j.jfoodeng.2017.05.005

[B106] Watte R. Aernouts B. Van Beers R. Postelmans A. Saeys W. (2016). Computational optimization of the configuration of a spatially resolved spectroscopy sensor for milk analysis. Anal. Chim. Acta 917, 53–63. doi: 10.1016/j.aca.2016.02.041 27026600

[B107] Wen X. Wang Z. Huang L. (2010). Measurement of myoglobin in pork meat by using steady spatially-resolved spectroscopy. J. Agric. Eng. 26, 375–379. doi: 10.3969/j.issn.1002-6819.2010.z2.071 21137423

[B108] Xia J. J. Berg E. P. Lee J. W. Yao G. (2007). Characterizing beef muscles with optical scattering and absorption coefficients in VIS-NIR region. Meat Sci. 75, 78–83. doi: 10.1016/j.meatsci.2006.07.002 22063414

[B109] Xia J. Weaver A. Gerrard D. E. Yao G. (2008). Distribution of optical scattering properties in four beef muscles. Sens. Instrument. Food Qual. Saf. 2, 75–81. doi: 10.1007/s11694-008-9032-6

[B110] Xie D. Guo W. (2020). Measurement and calculation methods on absorption and scattering properties of turbid food in Vis/NIR range. Food Bioproc. Technol. 13, 229–244. doi: 10.1007/s11947-020-02402-3

[B111] Yang W. Jin X. Gao X. (2021). Vector radiative transfer equation for arbitrary shape particles derived from Maxwell’s electromagnetic theory. J. Quant. Spectrosc. Radiat. Transf. 265, 107307. doi: 10.1016/j.jqsrt.2020.107307

[B112] Ye X. J. Doi T. Arakawa O. Zhang S. H. (2021). A novel spatially resolved interactance spectroscopy system to estimate degree of red coloration in red-fleshed apple. Sci. Rep. 11, 21982. doi: 10.1038/s41598-021-01468-z 34754021 PMC8578623

[B113] Yuan H. Liu C. Wang H. Wang L. Dai L. (2022). Early pregnancy diagnosis of rabbits: A non-invasive approach using Vis-NIR spatially resolved spectroscopy. Spectrochim. Acta Part A: Mol. Biomol. Spectrosc. 264, 120251. doi: 10.1016/j.saa.2021.120251 34455387

[B114] Zaytsev S. M. Amouroux M. Khairallah G. Bashkatov A. N. Tuchin V. V. Blondel W.. (2022). Impact of optical clearing on ex vivo human skin optical properties characterized by spatially resolved multimodal spectroscopy. J. Biophotonics 15, e202100202. doi: 10.1002/jbio.202100202 34476912

[B115] Zhang H. Hou Q. Luo B. Tu K. Zhao C. Sun Q. (2022). Detection of seed purity of hybrid wheat using reflectance and transmittance hyperspectral imaging technology. Front. Plant Sci. 13. doi: 10.3389/fpls.2022.1015891 PMC9554440 36247557

[B116] Zhang L. Tian H. Wang L. Li H. Pu Z. (2023). Selection and validation of the best detection location for hemoglobin determination by spatially resolved diffuse transmission spectroscopy. Infrared Phys. Technol. 133, 104839. doi: 10.1016/j.infrared.2023.104839

[B117] Zhang L. Wang Y. Bian H. Wang L. Li H. (2021). Optimal wavelengths selection from all points for blood species identification based on spatially resolved near-infrared diffuse transmission spectroscopy. Infrared Phys. Technol. 117, 103865. doi: 10.1016/j.infrared.2021.103865

[B118] Zhang G. W. Wen X. Wang Z. Y. Zhao D. J. Huang L. (2010). Measurement of pork tenderness by using steady spatially-resolved spectroscopy. Spectrosc. Spectral Anal. 30, 2793–2796. doi: 10.3964/j.issn.1000-0593(2010)10-2793-04 21137423

[B119] Zhao W. Zhao X. Luo B. Bai W. Kang K. Hou P.. (2023). Identification of wheat seed endosperm texture using hyperspectral imaging combined with an ensemble learning model. J. Food Compos. Anal. 121, 105398. doi: 10.1016/j.jfca.2023.105398

[B120] Zhou Y. Fu X. Ying Y. Fang Z. (2015). An integrated fiber-optic probe combined with support vector regression for fast estimation of optical properties of turbid media. Anal. Chim. Acta 880, 122–129. doi: 10.1016/j.aca.2015.04.048 26092344

[B121] Zhu Q. Guan J. Huang M. Lu R. Mendoza F. (2016). Predicting bruise susceptibility of ‘Golden Delicious’ apples using hyperspectral scattering technique. Postharvest Biol. Technol. 114, 86–94. doi: 10.1016/j.postharvbio.2015.12.007

[B122] Zhu Q. He C. Lu R. Mendoza F. Cen H. (2015). Ripeness evaluation of ‘Sun Bright’ tomato using optical absorption and scattering properties. Postharvest Biol. Technol. 103, 27–34. doi: 10.1016/j.postharvbio.2015.02.007

[B123] Zude M. Pflanz M. Spinelli L. Dosche C. Torricelli A. (2011). Non-destructive analysis of anthocyanins in cherries by means of Lambert–Beer and multivariate regression based on spectroscopy and scatter correction using time-resolved analysis. J. Food Eng. 103, 68–75. doi: 10.1016/j.jfoodeng.2010.09.021

